# COVID-19 mRNA vaccine-mediated antibodies in human breast milk and their association with breast milk microbiota composition

**DOI:** 10.1038/s41541-023-00745-4

**Published:** 2023-10-05

**Authors:** Shilin Zhao, Kris Y. W. Lok, Zhen Y. Sin, Ye Peng, Heidi S. L. Fan, Nitya Nagesh, Martha S. L. Choi, Jojo Y. Y. Kwok, Edmond P. H. Choi, Xi Zhang, Hogan Kok-Fung Wai, Leo C. H. Tsang, Samuel S. M. Cheng, Matthew K. L. Wong, Jie Zhu, Chris K. P. Mok, Siew C. Ng, Francis K. L. Chan, Malik Peiris, Leo L. M. Poon, Hein M. Tun

**Affiliations:** 1 Microbiota I-Center (MagIC), Hong Kong SAR, China; 2 Department of Medicine and Therapeutics, Faculty of Medicine, The Chinese University of Hong Kong, Hong Kong, Hong Kong SAR, China; 3 School of Nursing, The University of Hong Kong, Hong Kong, China; 4 The Jockey Club School of Public Health and Primary Care, Faculty of Medicine, The Chinese University of Hong Kong, Hong Kong SAR, China; 5 Li Ka Shing Institute of Health Sciences, Faculty of Medicine, The Chinese University of Hong Kong, Hong Kong SAR, China; 6 School of Public Health, Li Ka Shing Faculty of Medicine, The University of Hong Kong, Hong Kong SAR, China; 7 HKU-Pasteur Research Pole, LKS Faculty of Medicine, The University of Hong Kong, Hong Kong, China; 8 Li Ka Shing Institute of Health Sciences, State Key Laboratory of Digestive Disease, Institute of Digestive Disease, The Chinese University of Hong Kong, Hong Kong SAR, China; 9 Centre for Gut Microbiota Research, The Chinese University of Hong Kong, Hong Kong SAR, China

**Keywords:** RNA vaccines, RNA vaccines

## Abstract

Newborns can acquire immunological protection to SARS-CoV-2 through vaccine-conferred antibodies in human breast milk. However, there are some concerns around lactating mothers with regards to potential short- and long-term adverse events and vaccine-induced changes to their breast milk microbiome composition, which helps shape the early-life microbiome. Thus, we sought to explore if SARS-CoV-2 mRNA vaccine could change breast milk microbiota and how the changes impact the levels of antibodies in breast milk. We recruited 49 lactating mothers from Hong Kong who received two doses of BNT162b2 vaccine between June 2021 and August 2021. Breast milk samples were self-collected by participants pre-vaccination, one week post-first dose, one week post-second dose, and one month post-second dose. The levels of SARS-CoV-2 spike-specific IgA and IgG in breast milk peaked at one week post-second dose. Subsequently, the levels of both antibodies rapidly waned in breast milk, with IgA levels returning to baseline levels one month post-second dose. The richness and composition of human breast milk microbiota changed dynamically throughout the vaccination regimen, but the abundances of beneficial microbes such as *Bifidobacterium* species did not significantly change after vaccination. Additionally, we found that baseline breast milk bacterial composition can predict spike-specific IgA levels at one week post-second dose (Area Under Curve: 0.72, 95% confidence interval: 0.58–0.85). Taken together, our results identified specific breast milk microbiota markers associated with high levels of IgA in the breast milk following BNT162b2 vaccine. Furthermore, in lactating mothers, BNT162b2 vaccines did not significantly reduce probiotic species in breast milk.

## Introduction

Variants of the SARS-CoV-2 virus are causing significant disease burden worldwide, especially for unvaccinated children^1,2^. The vaccination of breastfeeding women against SARS-CoV-2 is recommended by health agencies to protect both the mother and infant^3–5^; SARS-CoV-2-specific antibodies can be found in both breast milk and infant stool post-vaccination^6,7^. However, in several countries, vaccination rates among lactating mothers are relatively low due to fears of unpredictable adverse effects on their infants^8–11^. This reluctance is a cause of concern, as breast milk antibodies not only confer protection to invading pathogens but can also shape the infant’s immune system development by facilitating the establishment and maintenance of the infants’ gut microbiome^12,13^. The early-life gut microbiome has a profound impact on long-term health^14,15^, including the establishment of T- and B-cell immunity^16^. Besides immune development, we previously found that the gut microbiota also plays a significant role in modulating the immune response to SARS-CoV-2 vaccines. *Bifidobactrium adolescentis* was found to be abundant in CoronaVac high-responders and associated with enriched carbohydrate metabolic pathways with immune-potentiating effects. *Prevotella copri* and two *Megamonas* species were suggested to play an anti-inflammatory role in reducing adverse events following BNT162b2 and CoronaVac vaccination^17^. However, the changes, if any, in breast milk microbiota composition post-vaccination and their influence on the antibody immune responses to SARS-CoV-2 vaccination in humans milk remains unelucidated. We therefore conducted a prospective longitudinal study in Hong Kong to investigate the change of microbiome composition before and after mRNA COVID-19 vaccination and how it impacts anti-SARS-CoV-2 antibody kinetics in the breast milk of lactating women.

## Results

### Recruitment of lactating mothers receiving BNT162b2

**Fig. 1:**
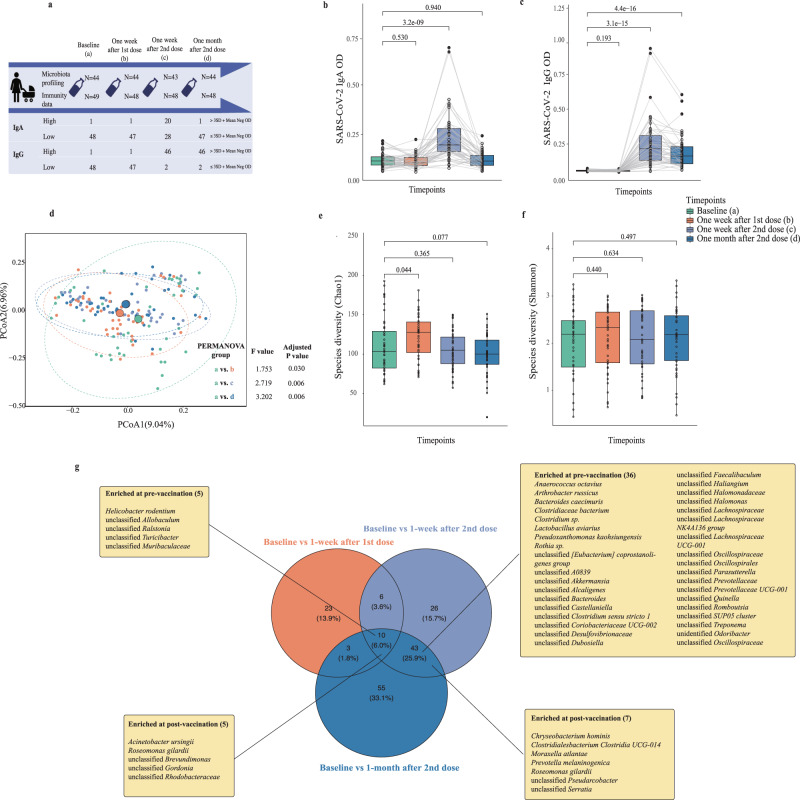
Study design and changes in immune responses to BNT162b2 vaccine, alpha diversity, and beta diversity in breast milk. **a** Study design. **b** Immunoglobulin (Ig)A–dominant humoral response detected in the breast milk of women who received BNT162b2. **c** Immunoglobulin (Ig)G–dominant humoral response detected in the breast milk of women who received BNT162b2. Comparison of levels of receptor-binding domain (RBD)–specific IgA (**B**) and IgG (**C**) in breast milk before and after vaccination. *p* values were given by Paired Wilcoxon rank-sum tests (two-sides) and adjusted for FDR. **d** Beta diversity was significantly different before and after completion of the vaccination regimen. p values were given by PERMANOVA and adjusted for FDR. **e** Alpha diversity based on species diversity (Chao1) pre- and post-vaccination*. p* values were given by linear mixed modeling adjusting by maternal age and time interval between two doses of vaccination (two-sided). **f** Alpha diversity based on species diversity (Shannon) pre- and post-vaccination*. p* values were given by linear mixed modeling adjusting by maternal age and time interval between the two vaccine doses (two-sided). **g** Venn diagram showing the trajectory of differential species between pre-vaccination and post-vaccination. The differential species were identified by the LEfSe (LDA score >1.5 and *p* value < 0.05). Elements on boxplots: centre line, median; box limits, upper and lower quartiles; whiskers, 1.5×IQR.

Between June and Dec 2021, 49 lactating mothers planning on receiving BNT162b2 were recruited and followed up at four timepoints (Fig. 1a). After removing samples without available sequencing data and one outlier (positive ELISA result pre-vaccination), 175 samples from 44 participants remained for downstream analyses. Participants’ ages ranged from 25 to 42 years, with a median age of 36 years (interquartile range (IQR): 31, 39). The samples were collected at a median lactation stage of 36.64 weeks (IQR: 20.32, 47.29). Nearly half (44.2%) of the participants gave birth via planned cesarean section. The intervals between two vaccine doses ranged between 20 – 42 days (median: 21 days, IQR: 21, 27) (Table 1).

**Table 1:** Baseline characteristics of the study population in different immunity response group at one week after second dose.

Variables	Overall (*N* = 43)	Low-IgA subjects (*N* = 25)	High-IgA subjects (*N* = 18)	*P* value
Maternal age (years)				0.250
Median (IQR)	36.0 (31.0,39.0)	37.0 (32.0,39.0)	32.0 (30.3,37.8)	
Gestational age (weeks)				0.960
Median (IQR)	38.9 (38.0, 39.7)	38.9 (38.0, 39.7)	38.9 (38.0, 39.8)	
Lactation stage at baseline (weeks)				0.971
Median (IQR)	36.6 (20.3, 47.3)	36.6 (26.3, 45.9)	35.9 (17.2, 57.3)	
Infant’s birth weight (kg)				0.658
Median (IQR)	3.2 (3.1, 3.6)	3.2 (3.0, 3.6)	3.2 (3.0, 3.6)	
Total number of previous children				0.213
0	3 (7.0)	3 (12.0)	0 (0)	
1	20 (46.5)	12 (48.0)	8 (44.4)	
2	15 (34.9)	6 (24.0)	9 (50.0)	
3	5 (11.6)	4 (16.0)	1 (5.6)	
Delivery type				0.414
Spontaneous vaginal delivery	13 (30.2)	5 (20.0)	8 (44.4)	
Assisted vaginal delivery	3 (7.0)	2 (8.0)	1 (5.6)	
Planned C-section	19 (44.2)	13 (52.0)	6 (33.3)	
Emergency C- section	8 (18.6)	5 (20.0)	3 (16.7)	
Labor induced				1
No	28 (65.1)	16 (64.0)	12 (66.7)	
Yes	15 (34.9)	9 (36.0)	6 (33.3)	
Epidural anesthesia use				0.057
No	25 (58.1)	11 (44.0)	14 (77.8)	
Yes	17 (39.5)	13 (52.0)	4 (22.2)	
Missing	1 (2.3)	1 (4.0)	0 (0)	
Intramuscular analgesia use				0.516
No	31 (72.1)	19 (76.0)	12 (66.7)	
Yes	12 (27.9)	6 (24.0)	6 (33.3)	
Infant’s sex				0.765
Female	25 (58.1)	14 (56.0)	11 (61.1)	
Male	18 (41.9)	11 (44.0)	7 (38.9)	
Smoked previously				0.419
No	42 (97.7)	25 (100)	17 (94.4)	
Yes	1 (2.3)	0 (0)	1 (5.6)	
Partner smokes				1
No	38 (88.4)	22 (88.0)	16 (88.9)	
Yes	5 (11.6)	3 (12.0)	2 (11.1)	
Marital status				0.419
Married	42 (97.7)	25 (100)	17 (94.4)	
Not married	1 (2.3)	0 (0)	1 (5.6)	
Place of birth				0.766
Hong Kong SAR	33 (76.7)	19 (76.0)	14 (77.8)	
Mainland, China	6 (14.0)	3 (12.0)	3 (16.7)	
Others	4 (9.3)	3 (12.0)	1 (5.6)	
Years living in HK (customized)				0.455
<15 years	9 (20.9)	4 (16.0)	5 (27.8)	
> = 15 years	34 (79.1)	21 (84.0)	13 (72.2)	
Educational level (customized)				0.47
Postgraduate degree or above	14 (32.6)	9 (36.0)	5 (27.8)	
Below university level	8 (18.6)	3 (12.0)	5 (27.8)	
University degree	21 (48.8)	13 (52.0)	8 (44.4)	
Family income per month (customized)				0.144
HK$40,000 or more	38 (88.4)	24 (96.0)	14 (77.8)	
Under HK$40,000	5 (11.6)	1 (4.0)	4 (22.2)	

Categorical data are presented as number (percentage) and continuous variables as median (interquartile range). Within-group valid percentages are shown. Only subjects with both available antibody and 16 S data (at one week after second dose) were summarized in the table. *P* values were given by Fisher’s exact test for the categorical variables and Wilcoxon rank-sum test for continuous variables.

### Levels of anti-SARS-CoV-2 IgA and IgG in breast milk were boosted after two vaccine doses

After the first dose, levels of SARS-CoV-2 spike protein-specific IgA and IgG in breast milk remained unchanged compared with pre-vaccination. Levels were boosted one week post-second dose (median (IQR), IgA OD: 0.116 (0.096, 0.137) vs. 0.198 (0.164, 0.280), Fig. 1b; IgG OD: 0.058 (0.054, 0.062) vs. 0.216 (0.132, 0.310), *p* < 0.001, Fig. 1c, Paired Wilcoxon rank-sum tests). Although IgA levels returned to baseline levels one month post-second dose (Paired Wilcoxon rank-sum tests*, p* < 0.001), IgG levels remained significantly higher compared to baseline (Paired Wilcoxon rank-sum tests, IgG OD: 0.058 vs. 0.164, *p* < 0.001). Additionally, there was a significant moderate association between the two antibodies in breast milk (Spearman’s rho coefficient: 0.46*, p* < 0.001 Supplementary Fig. 1a).

We also found that mothers who received epidural anesthesia during delivery had a lower IgA levels one week post-second dose (Mann-Whitney tests, *p* = 0.020, Supplementary Table 1).

### Changes in breast milk bacterial richness and composition were observed post-vaccination

We conducted 16 S rRNA amplicon sequencing analysis to determine if microbiome composition was associated with levels of vaccine-induced anti-RBD antibodies in breast milk. Breast milk microbial composition (Supplementary Fig. 2a) and diversity (alpha and beta diversities) (Fig. 1d–f) were compared over different timepoints. We observed a significant increase in bacterial Chao1 richness between pre-vaccine and one week post-first dose samples *(p* = 0.044, Linear mixed model), but not in terms of Shannon diversity *(p* = 0.440, Fig. 1e, f, Linear mixed model). Microbiome richness returned to baseline levels one week post-second dose. To determine whether this change in alpha diversity is associated with vaccination, we analyzed longitudinal breast milk microbiota data from an independent, unvaccinated cohort^18^, in which we observed a continuous decline of Chao1 diversity (Supplementary Fig. 1b). Regarding beta-diversity, a significant shift was observed between pre-vaccination and all post-second dose vaccination timepoints (adjusted *p* = 0.030, 0.006, and 0.006 for one week post-first dose and one week and one month post-second dose, respectively, Fig. 1d, pairwise Adonis test). However, no significant changes in microbiome beta diversity were observed in the independent cohort (Supplementary Fig. 1c; adjusted *p* = 0.240 0.360, 1.000, respectively, pairwise Adonis test). Intramuscular analgesia during labor was significantly associated with baseline breast milk microbiome composition, but it showed no association with IgA levels post-second vaccination (PERMANOVA, *p* = 0.012 and 0.807, respectively, Supplementary Table 1-2). Additionally, the lactation stage did not significantly influence the beta-diversity of baseline human milk microbiota (PERMANOVA, *p* = 0.349, Supplementary Table 2).

We then conducted LEfSe to identify differentially abundant bacterial taxa between timepoints. In total, 109 differentially abundant species were identified between pre- and post-vaccination timepoints, ten of which remained differentially abundant throughout all three post-vaccination timepoints. Fifty-three species were differentially abundant between baseline levels and post-second dose, including *Anaerococcus octavius*, *Arthrobacter russicus*, *Bacteroides caecimuris*, *Clostridiaceae bacterium, Helicobacter rodentium*, *Lactobacillus aviarius*, and *Rothia sp*. (LEfSe, LDA score >1.5 and *p* value < 0.05, Fig. 1g, Supplementary Table 3). The 10 most abundant species varied across samples and timepoints (Supplementary Fig. 2).

### Baseline breast milk bacterial composition is associated with anti-SARS-CoV-2 antibody levels in breast milk following vaccination

We then investigated whether breast milk bacterial composition was associated with its antibody levels post-vaccination. Since immune parameters peaked at one week post-second dose (Fig. 1b, c), we focused our subsequent analyses on this timepoint, thereby dichotomizing the cohort into those with high- and low-IgA levels using the prior-specified cutoff (High, 20; Low, 28; Fig. 1a).

**Fig. 2:**
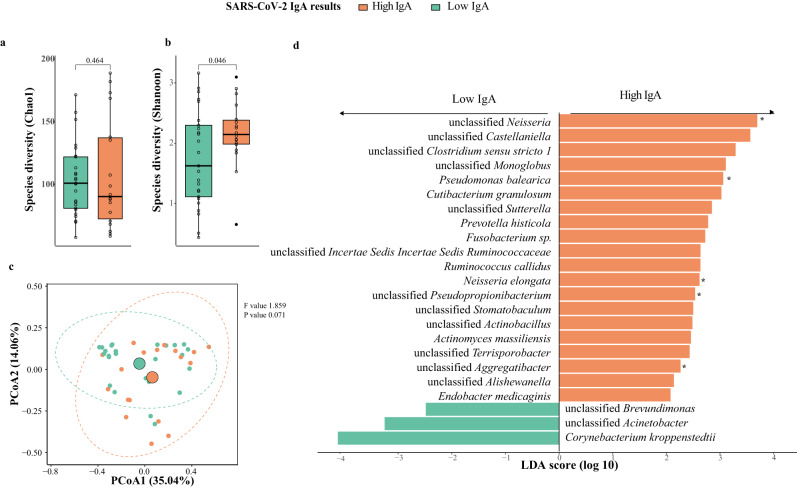
Baseline breast milk microbiota composition in mothers with high and low responses at one week post-second dose of BNT162b2 (*N* = 43; High IgA: 18, Low IgA: 25). **a** Bacteria diversity. **b** Bacteria richness. *P* values were given by Wilcoxon rank-sum test. **c** Principal coordinates analysis (PCoA) of breast milk microbiota composition of mothers with high- and low-IgA levels at one week post-second dose BNT162b2. *p* value was given by PERMANOVA. **d** Linear discriminant analysis effect size analysis of discriminant taxa in baseline breast milk microbiome of mothers with high and low-IgA levels at one week post-second dose of BNT162b2. LDA linear discriminant analysis. Elements on boxplots: centre line, median; box limits, upper and lower quartiles; whiskers, 1.5×IQR.

The Chao1 richness and beta diversity of baseline microbiota were not significantly different between high- and low-IgA participants (Wilcoxon sum-rank test, *p* = 0.464 and PERMANOVA, 0.071, respectively, Fig. 2a). However, the baseline Shannon richness in high-IgA subjects was significantly higher than that of low-IgA subjects (Wilcoxon rank-sum test, *p* = 0.046, Fig. 2b).

We then investigated whether there were taxonomic differences in baseline microbiota between high- and low-IgA subjects. For both groups, the most abundant phylum was Firmicutes, followed by Proteobacteria and Actinobacteria (Supplementary Fig. 3). The relative abundance of Firmicutes was higher in low-IgA subjects (median relative abundance of 61.78% and 74.62% for high- and low-IgA group, respectively), while levels of Proteobacteria were depleted (18.64% and 9.01% for high- and low-IgA group, respectively, Supplementary Fig. 3).

We identified 23 differentially abundant species between the high- and low-IgA subjects (LEfSe, *p* = 0.004, Fig. 2d). Unclassified *Neisseria* and *Corynebacterium kroppenstedti* showed the largest effect size in enrichment for high- and low-IgA subjects, respectively (LEfSe, LDA score: 3.69 and 4.12, respectively). The mixed effects model showed that unclassified *Neisseria* and *Neisseria elongata* were persistently higher in subjects with high breast milk IgA levels (*p* = 0.045 and 0.031, respectively, Supplementary Table 4). The lactation stage did not impact the relative abundance of these markers, including unclassified *Neisseria*, *Neisseria elongate*, and probiotic species (Supplementary Fig. 4).

**Fig. 3:**
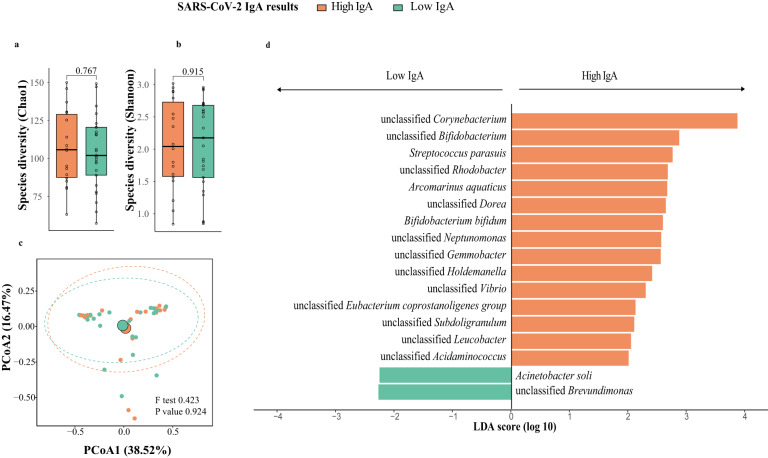
Breast milk microbiota composition in mothers with high and low responses at one week post-second dose of BNT162b2 (*N* = 43; High IgA: 18, Low IgA: 25). **a** Bacteria diversity. **b** Bacteria richness. *P* value comparing the diversity and richness were given by Wilcoxon rank-sum test. **c** Principal coordinates analysis (PCoA) of breast milk microbiota composition of mothers with high- and low-IgA levels at one week post-second dose of BNT162b2. *p* value was given by PERMANOVA. **d** Linear discriminant analysis effect size analysis of discriminant taxa in breast milk microbiome of mothers with high- and low-IgA levels at one week post-second dose of BNT162b2. LDA linear discriminant analysis. Elements on boxplots: centre line, median; box limits, upper and lower quartiles; whiskers, 1.5×IQR.

Besides differences in baseline microbiota, taxonomic differences in microbiota one week post-second dose between high- and low-IgA subjects were identified by LEfSe. Higher abundances of *Bifidobacterium bifidum* and unclassified *Bifidobacterium* were found in the high-IgA subjects (LDA score: 2.59 and 2.87, respectively, Fig. 3).

**Fig. 4:**
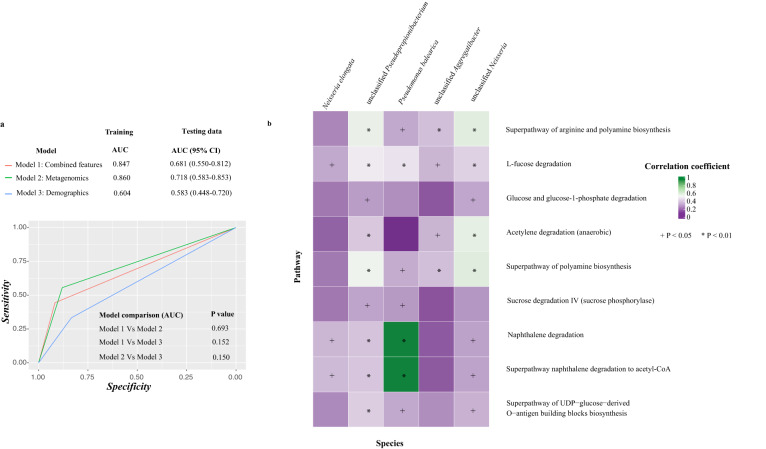
Baseline breast milk bacterial species and functions associated with high- and low-IgA participants to BNT162b2 vaccination at one week post-second vaccine dose (*N* = 43; High IgA: 18, Low IgA: 25). **a** AUROC (95% CI) values of models based on metagenomic markers, demographical markers (maternal age, different time interval between two doses of vaccine, and the use of epidural anesthesia), and combined markers using a random forest classification approach. AUROC, area under the receiver operating characteristic curve. The performance of random forest models was compared using the bootstrap method. **b** Baseline bacterial species and pathways associated with levels of immunoglobulin A. Differential baseline breast milk bacterial species and pathways were detected by a random forest model and Wilcoxon’s rank-sum test (two-sided), respectively. P values were given by Spearman’s correlation test.

We then used random forest to investigate the power of these distinct microbial species in predicting levels of anti-SARS-CoV-2 antibodies in breast milk one week post-second dose. We constructed a model based on five microbial features including *Neisseria elongata*, *Pseudomonas balearica*, unclassified *Pseudopropionibacterium*, unclassified *Neisseria*, and unclassified *Aggregatibacter*. The model based solely on microbial features [AUC (95% CI): 0.72 (0.58-0.85)] performed better than the model which combined microbial and demographic predictors [AUC (95% CI): 0.68 (0.55-0.81), Fig. 4a, *p* value: 0.693, bootstrap test].

To investigate the potential mechanisms by which pre-vaccination breast milk microbiota affects antibody levels, we estimated the association between predicted microbial functional pathways and anti-SARS-CoV-2 IgA levels at one week post-second dose. A total of 437 pathways were predicted by PICRUSt2, among which nine were significantly enriched in high-IgA subjects (Wilcoxon rank-sum, adjusted *p* < 0.05, Fig. 4). The correlation network showed that higher abundances of selected bacterial markers were associated with sugar metabolism pathways (e.g., sucrose degradation IV, glucose and glucose-1-phosphate degradation, fucose degradation). For example, fucose degradation was positively correlated with all bacterial markers. Additionally, superpathways for arginine and polyamine biosynthesis were correlated with all markers expect for *Neisseria elongate* (Fig. 4b).

### Impact of vaccination on the abundance of *Lactobacilli* and *Bifidobacteria* in breast milk

**Fig. 5:**
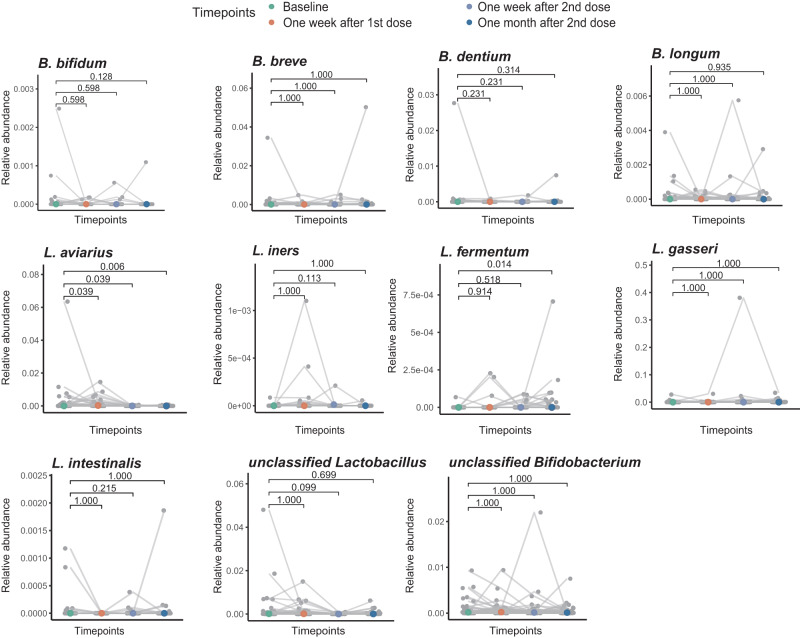
Relative abundance of *Bifidobacterium* and *Lactobacillus* in breastmilk at different timepoints (Sample size, Baseline: 44, One week after 1st dose: 44, One week after 2nd dose: 43, One month after second dose: 44). The median value was shown in different colors by different timepoints. *p* values were given by Paired Wilcoxon rank-sum tests (two-sides) and adjusted for FDR.

*Lactobacilli* and *Bifidobacteria* constitute a core microbiota of shared taxa between breast milk and infant stool^19,20^. Given those genera play a beneficial role in the healthy development of the infant’s gut microbiota and immune system^21,22^, we examined the association between these taxa and immune responses to BNT162b2 in breast milk. We detected the presence of *Lactobacilli* (*L. aviarius*, *L. fermentum*, *L. gasseri*, *L. iners*, *L. intestinalis*, and unclassified *Lactobacillus*) and *Bifidobacteria* (*B. bifidum*, *B. breve*, *B. dentium*, *B. longum*, and unclassified *Bifidobacterium*). Vaccination did not significantly change the abundance of *Lactobacillus* and *Bifidobacterium* in breast milk, except for *L. aviarius* and *L. ferment*um (Fig. 5, Supplementary Table 5). However, these two genera were not persistently high in subjects with high IgA levels (Supplementary Table 6).

## Discussion

Breast milk vertically transmits maternal antibodies and microbes to infants^12,23^. To the best of our knowledge, this is the first longitudinal human study to show that baseline breast milk microbiota composition and its post-vaccination changes reflect vaccine-conferred anti-SARS-CoV-2 antibody levels in breast milk. The post-vaccination changes in bacterial richness differ from those in the external longitudinal cohort of unvaccinated lactating mothers, suggesting that these changes are attributable to BNT162b2 vaccination. Vaccination did not significantly impact the relative abundances of the beneficial *Bifidobacteria*. We also identified differential abundant bacterial species at the baseline timepoint which were associated with higher immune responses. For example, unclassified *Neisseria* and *Neisseria elongata* were enriched at pre-vaccination in participants with higher anti-SARS-CoV-2 antibody levels in their breast milk post-vaccination.

We found at least three ways in which microbiota are associated with anti-SARS-CoV-2 antibody levels. Firstly, unclassified *Neisseria* and *Neisseria elongata* were consistently enriched at pre-vaccination in mothers with higher IgA levels in their breast milk post-vaccination. These immunomodulatory *Neisseria sp*. may increase anti-SARS-CoV-2 antibody levels by interacting with T- and B-cells^24^. These species can also mediate inflammatory responses through toll-like receptor 2^24^. The outer membrane vesicles of these species could therefore act as adjuvants to boost BNT162b2 immunogenicity by enhancing IgG immune responses^25^. However, the safety profile of these species and causality need to be established in further experiments.

The interaction between breast milk microbiota and antibody levels could also be a result of the microbiota’s influence on immunomodulatory metabolic pathways. Our data indicated that higher abundances of selected bacterial markers are associated with metabolic pathways (e.g., fucose degradation), the latter of which may enhance vaccine-induced immunity levels in breast milk. Nine microbial metabolic pathways were enriched in high-IgA subjects, including fucose degradation and super pathways responsible for arginine and polyamine biosynthesis. Most of these pathways, especially the sugar metabolism-related pathways, could play immunomodulatory roles (e.g., inflammation and T-cell modulation) once transmitted from breast milk to the infant gut^26–29^. For example, sucrose degradation IV (sucrose phosphorylase) (PWY-5384) and glucose and glucose-1-phosphate degradation (GLUCOSE1PMETAB-PWY) pathways were the most significant pathways enriched in newborn feces compared to breast milk, with the former pathway being found in the *Bifidobacterium* genus^20^. Glucose and glucose-1-phosphate degradation metabolites are used by *Enterobacteriaceae* for immediate energy and may induce an inflammatory response^28^. As such, the breast milk microbiota could favor the immune response to BNT162b2 by modulating sugar metabolism-related pathways.

The enrichment of fucose degradation pathways, which are immunomodulatory^30^ and can be utilized by *Bifidobacterium* species^31–33^, in high-IgA subjects indicates the importance of breast milk metabolite composition in shaping the immune response. Fucose, a key component of human milk oligosaccharides (HMOs) which plays a vital role in infant gut colonization and development of innate immunity during early life^34–36^, may be utilized by gut commensals to facilitate colonization^37^ and regulate SCFA production by modulating mucosal immune components^30,38–40^. This increased SCFA production has been linked with enhanced systemic immune responses after cholera and influenza vaccination in mice^40^. Additionally, fucosylation of sIgA contributes to intestinal proteolytic resistance and binding to pathogenic bacteria^35,41^. Therefore, SARS-CoV-2 vaccination may also affect immune responses through breast milk metabolite composition.

The increased abundances of the probiotic *Bifidobacteria* may also be linked to increased anti-SARS-CoV-2 antibody levels. Both the abundances of unclassified *Bifidobacterium* and *B. bifidum* at the 1-week post-second dose timepoint were significantly higher in subjects with higher IgA levels. As *Bifidobacterium* species can utilize L-fucose and exert acceptive immunity^32^, the enrichment of fucose degradation pathways in high-IgA subjects could be attributed to a higher preference of *Bifidobacterium* species in this group. Notably, the abundances of these taxa were not affected by vaccination as they were not significantly different between pre- and post-vaccination. As such, these taxa might have potential immunological benefits, which have also been observed in prior intervention studies. Previous studies used *Bifidobacteria* probiotics to successfully enhance vaccine efficacy (e.g., against influenza, diphtheria, rotavirus, and polio), with increased humoral responses following the vaccination of neonates and young children^40,42^. Infant gut *Bifidobacteria* abundance is also associated with CD4^+^ T-cell responses and increased antibody responses to *Bacillus* Calmette-Guérin, tetanus toxoid, and hepatitis B virus vaccines^43,44^. These *Bifidobacteria*-associated effects may therefore extend to the SARS-CoV-2 vaccine. Further downstream longitudinal studies focused on infants and vaccine efficacy are required to assess how the probiotic microbiota in breast milk influence immune responses in infants.

SARS-CoV-2 mRNA vaccine induces changes in breast milk microbiota, including bacterial richness and composition, whereas the impacts of SARS-CoV-2 infection remain mixed. A study conducted by Natalia et al., collected breast milk samples from 37 pregnancies with mild SARS-CoV-2 infection and 63 healthy mothers during the first and fifth postpartum weeks. Interestingly, they revealed no distinct bacterial profiles and alpha diversity between milk samples from the case and control groups. This finding suggested that mild SARS-CoV-2 infection during the early postpartum period might not substantially impact the composition and diversity of breast milk microbiota^45^. Juárez-Castelán et al., collected human colostrum samples and rectal swabs with the presence of SARS-CoV-2 virus. They discovered that nearly 20% of the bacterial diversity found in human colostrum was also present in the mother’s rectal swab samples. This finding suggests a potential influence of gut microbiota on the microbiota of breast milk. This influence could be attributed to the translocation of internal bacteria from the mother’s gastrointestinal (GI) tract to the mammary gland, a process that is facilitated by immune cells and typically occurs during the late stages of pregnancy^46^. In our previous research, we observed that the SARS-CoV-2 mRNA vaccine can induce gut microbiota dysbiosis^17^. As such, the gut microbiota dysbiosis triggered by the SARS-CoV-2 mRNA vaccine could contribute to alterations in the breast milk microbiota following vaccination. On the other hand, the changes induced by the SARS-CoV-2 vaccine in breast milk microbiota imply a different immunological response that may be distinct from that triggered by a natural infection. Additionally, variations in the findings across studies might be due to differences in the methodologies used, the timing of sample collection, and medication consumption in the mothers.

Changes in breast milk post SARS-CoV-2 vaccination may confer immunological protection to infants via two distinct mechanisms: through antibodies and microbiota. Regarding protection conferred by the former, elevated levels of anti-SARS-CoV-2 antibodies in breast milk could provide barrier immunity and protect infants by coating the surface of their gastrointestinal tract^47,48^. We found increased levels of anti-SARS-CoV-2 IgA and IgG in the breast milk of mothers after two doses of BNT162b2-vaccination. However, IgA levels decreased one week post-second dose while IgG levels remained high, which is consistent with prior studies^49–52^. Since SARS-CoV-2 antibodies can be transferred to infants through breast milk^7^, the waning of IgA levels over time may decrease levels of breast milk-borne anti-SARS-CoV-2 antibodies transferred to infants from breast milk and, by extension, immunological protection to SARS-CoV-2.

A strength of this study was the longitudinal assessment of breast milk microbiota and complete breast milk antibody data collection pre- and post-vaccination. However, the study was limited by its small sample size and lack of a longitudinal control cohort without COVID-vaccination, due to difficulties in recruiting subjects during the pandemic. Nevertheless, this limitation was made up through including an external validation cohort, albeit samples were collected pre-pandemic and the lactation stages did not align. However, we believe this control cohort is still valid, given that lactation stage did not significantly impact microbiota compositions (Supplementary Table 2), and this control cohort was the closest in collection intervals and ethnicities. Ideally, larger cohorts across different populations, as well as a control cohort collected during the pandemic are needed to validate our findings. We did not measure neutralizing antibody levels in breastmilk. However, our findings are still valid, as prior studies reported a moderate positive correlation between levels of neutralizing antibody and SARS-CoV-2-specific antibodies at one week post-second dose^53^. Besides, further investigations are necessary to elucidate the safety profile, the immunomodulatory role of *Neisseria sp*. as a BNT162b2 adjunct, and the mechanisms by which fucosylation shapes the breast milk microbiome. Moreover, as clinical and metagenomic data from infants were not collected, we could not determine the infants’ immunity to SARS-CoV-2 and how breast milk microbiota was transmitted to the infant gut. The role of this transmission in immunomodulation could not be explained quantitively by our study. Additionally, the levels of oligosaccharides, a potential modulator of microbiota composition, were not measured, which may act as bias in the study^54^. Future studies incorporating this parameter would be beneficial to fully elucidate the interaction between oligosaccharides, microbiota composition, and the immune response to vaccination. Lastly, the effects of vaccines based on different mechanisms, such as CoronaVac, remains to be investigated.

In summary, we observed several changes in breast milk microbiota post-BNT162b2 vaccination, except for *Bifidobacteria* abundances. The abundances of probiotic species were significantly associated with vaccine-induced immunity levels in breast milk. Vaccination of lactating mothers may therefore impart additional immunological protection to infants through breast milk by both the vertical transmission of antibodies and beneficial microbiota.

## Methods

### Prospective cohort recruitment and sample collection

We recruited 49 lactating mothers who planned to receive the BNT162b2 mRNA vaccine, when more than 99% of the Hong Kong population was not infected by COVID-19 (late 2021)^55^. Mothers who were serologically negative for SARS-CoV-2 pre-vaccination were included, while those who failed to receive two doses of BNT162b2 were excluded. Breast milk was self-collected at the participants’ home in sterile milk bags at four timepoints (pre-vaccination, one week post-first dose, one week post-second dose, and one month post-second dose, Fig. 1). Samples were sent to the laboratory within eight hours of collection, aliquoted, and preserved at -80 °C until DNA extraction. Demographic and lifestyle factors including age, education, and breastfeeding habits, were collected through self-administered questionnaires at each timepoint. The study protocol was approved by the University of Hong Kong/Hospital Authority Hong Kong West Cluster Institutional review board (IRB Ref. No. UW-21-415). All participants provided written consent during enrolment. This study followed the STROBE reporting guideline.

### ELISA for SARS-CoV-2 spike protein-specific IgA and IgG

SARS-CoV-2 spike protein-specific IgA and IgG levels in breast milk were measured to assess immune responses to BNT162b2 at different stages of the vaccination regimen. Breast milk samples were centrifuged for 15 min at 1000 x *g* at 2–8 °C and the aqueous fraction was collected. This process was repeated twice. The samples were then immediately tested for SARS-CoV-2 spike receptor-binding domain-specific (RBD-specific) IgG or IgA using ELASA assay^56^. 96-well ELISA plates (Nunc MaxiSorp, Thermo Fisher Scientific) were coated overnight with 100 ng per well of the purified recombinant RBD protein in phosphate-buffered saline (PBS) buffer. Concurrently, a control plate was coated solely with PBS buffer overnight. This control served to subtract non-specific binding to the plate, i.e. acting as a normalization method for serum-specific background noise (SSBN). The plates coated with either purified recombinant protein or PBS were then blocked with 100 μl of Chonblock blocking/sample dilution ELISA buffer (Chondrex Inc, Redmon, US) at room temperature for 2 h. Each breast milk sample was tested at a dilution of 1:100 in Chonblock blocking/sample dilution ELASA buffer and added to the ELISA plate wells, incubated for 2 h at 37 °C, and then extensively washed with PBS containing 0.1% Tween 20. Following the extensive washing, horseradish peroxidase (HRP)-conjugated goat anti-human IgG (Thermo Fisher Scientific, US, Cat. No. 31410) (1:5000) or HRP-conjugated goat anti-human IgA (Thermo Fisher Scientific, US, Cat. No. PA1-74395) (1:5000) by incubating for 1 h at 37 °C.

Post incubation, ELISA plates were washed five times with PBS containing 0.1% Tween 20. Subsequently, 100 μL of HRP substrate (Ncm TMB One; New Cell and Molecular Biotech Co. Ltd, Suzhou, China, Cat. No. M30500) was added into each well. After a 15-minute incubation, the reaction was halted with 50 μL of 2 M H2SO4 solution. The absorbance was measured using a Sunrise (Tecan, Männedorf, Switzerland) absorbance microplate reader at a 450 nm wavelength. The cutoff point of antibody levels was derived from the mean optical density (OD) plus three standard deviations of the negative controls (Fig. 1a). IgA levels were used as the main outcome for subsequent analyses due to its predominance and significant immune functionality in breast milk^57^.

### DNA extraction from breast milk samples

Breast milk was first pre-processed according to Douglas et al.^58^. The DNA in the cell pellet was then extracted using QIAamp PowerFecal Pro DNA Kit (QIAGEN, Hilden, Germany, Cat. No. 51804). Briefly, 3 mL of breast milk sample was centrifuged at 13,000 x *g* for 20 min. The separated fat layer and liquid supernatant were then removed. The DNA in the cell pellet was then extracted using QIAamp PowerFecal Pro DNA Kit (QIAGEN, Hilden, Germany) according to the manufacturer’s instructions, which included a bead-beating step. DNA concentration was measured using a NanoDrop™ ND-1000 spectrophotometer (NanoDrop Technologies, DE, USA).

### QIIME2 pipeline

Briefly, the raw sequence reads were quality checked, demultiplexed into FASTQ files, denoised using DADA2 (q2-dada2 denoise-paired), and subsampled. Taxonomic IDs were then assigned to amplicon sequence variants (ASVs) against the silva-138-99-nb-classifier for taxonomic output. The ASV profiles were rarefied to 60,407 reads per sample. A total of 2116 ASVs in the vaccine cohort and 1457 ASVs in the control cohort remained after filtering out those with at least 2 reads and ASVs which occurred in less than 2% of participants. Alpha diversity indices (within-sample diversity), including observed ASVs and Chao1 richness, were then calculated. Beta diversity (between-sample diversity) was determined by calculating Bray-Curtis distance.

### Breast milk microbiota analysis

DNA was extracted from breast milk using QIAamp PowerFecal Pro DNA Kit (QIAGEN, Hilden, Germany). V3-V4 regions of the 16 S rRNA genes were amplified and sequenced by Illumina MiSeq. The QIIME2 pipeline^59^ was used to generate amplicon sequence variants (ASVs). ASV profiles were summarized into phylum- to species-level taxonomic profiles. Sequencing reads were deposited at Sequence Read Archive under accession PRJNA917338. The same pipeline was applied to the validation cohort^18^, which consists of 110 Chinese lactating mothers without COVID19-vaccination. Their breast milk samples were collected at 4, 8, and 12 weeks (T1, T2, and T3) postpartum. Microbial functional profiles of the breast milk samples were predicted using PICRUSt2^60^.

### Statistical analyses

Continuous variables were summarized by reporting their median (IQR), while categorical variables were presented as a percentage of the cohort. Qualitative and quantitative differences between subgroups were analyzed using Fisher’s exact tests and Mann-Whitney tests for categorical and continuous parameters, respectively. We primarily explored the association between breast milk microbiota composition and levels of SARS-CoV-2 spike receptor-binding domain-specific antibodies.

To assess the effect of timepoints on α-diversity, linear mixed modeling was conducted. Alpha-diversity indices were regressed against time and adjusted for maternal age and time intervals between the two vaccine doses. Individual identifier numbers were modeled as a random effect. Principal coordinates analysis (PCoA) was used to visualize sample clustering based on species-level compositional profiles. Pairwise multilevel comparisons across different timepoints were carried out on the Bray-Curtis distance matrix using pairwise adonis test.

Comparisons of *Lactobacilli* and *Bifidobacteria abundances* across different timepoints were done using Dunn’s test with false discovery rate (FDR) correction. Differential microbial markers between responder groups were identified using linear discriminant analysis (LDA) effect size (LEfSe) with an LDA score > 2 and a *p* value < 0.05. Multivariate analysis by the linear models (MaAsLin2) statistical framework was implemented to evaluate the association between specific microbial markers and demographical characteristics in the Huttenhower Lab Galaxy instance (http://huttenhower.sph.harvard.edu/galaxy/). The *p* values for metagenomics analyses were adjusted for multiple comparisons using FDR correction.

To predict levels of anti-SARS-CoV-2 antibodies in breast milk one week post-second dose using baseline breast milk microbiota abundances, classification models were constructed using a random forest approach. Two models were constructed: one based on significant differential microbial predictors and one which combined these microbial predictors with demographic factors (age, time interval between vaccination, and intramuscular analgesia use during labor). The model was tested by a leave-one-out approach. The pROC package in R was then used to calculate the area under the curve (AUC) of receiver operator characteristic (ROC) curves and the corresponding 95% confidence interval (CI) for each model. The performance of random forest models was compared using the bootstrap method. Unless otherwise specified, all analyses and data visualization were performed in R V4.2.0. Two-sided values of *p* values < 0.05 were considered statistically significant.

### Reporting summary

Further information on research design is available in the Nature Research Reporting Summary linked to this article.

### Supplementary information

Supplementary Materials

Reporting Summary

## Data Availability

Sequencing reads were deposited at Sequence Read Archive under accession PRJNA917338 on the National Center for Biotechnology Information. Additional datasets generated and/or analysed in this study are available from the corresponding author on reasonable request.

## Code availability

The underlying code for this study is not publicly available but may be made available to qualified researchers on reasonable request from the corresponding author.

## References

[1] Khandia R (2022). Emergence of SARS-CoV-2 Omicron (B.1.1.529) variant, salient features, high global health concerns and strategies to counter it amid ongoing COVID-19 pandemic. Environ. Res..

[2] Nathanielsz, J., Toh, Z. Q., Do, L. A. H., Mulholland, K. & Licciardi, P. V. SARS-CoV-2 infection in children and implications for vaccination. *Ped. Res.*10.1038/s41390-022-02254-x (2022). 10.1038/s41390-022-02254-x PMC9376896 35970935

[3] Medicine, A. O. B. *Considerations for COVID-19 Vaccination in Lactation*, https://www.bfmed.org/abm-statement-considerations-for-covid-19-vaccination-in-lactation Accessed Feburary 16, 2023 (2020).

[4] Gynecologists, T. A. C. O. O. A. *COVID-19 Vaccination Considerations for Obstetric–Gynecologic Care*. https://www.acog.org/clinical/clinical-guidance/practice-advisory/articles/2020/12/covid-19-vaccination-considerations-for-obstetric-gynecologic-care Accessed Feburary 16, 2023 (2023).

[5] Prevention, C. F. D. C. A. *COVID-19 Vaccines While Pregnant or Breastfeeding*, https://www.cdc.gov/coronavirus/2019-ncov/vaccines/recommendations/pregnancy.html Accessed Feburary 16, 2023 (2022).

[6] Pietrasanta C (2022). Humoral response to anti-SARS-CoV-2 vaccine in breastfeeding mothers and mother-to-infant antibody transfer through breast milk. npj Vaccines.

[7] Low JM (2021). Codominant IgG and IgA expression with minimal vaccine mRNA in milk of BNT162b2 vaccinees. npj Vaccines.

[8] Knight H (2021). Understanding and addressing vaccine hesitancy in the context of COVID-19: development of a digital intervention. Public Health.

[9] Razzaghi H (2022). COVID-19 vaccination coverage and intent among women aged 18-49 years by pregnancy status, United States, April-November 2021. Vaccine.

[10] Halemani K, Dhiraaj S, Latha T, Mishra P, Issac A (2022). The prevalence of COVID vaccine acceptance among pregnant women: A systematic review and meta-analysis. Clin. Epidemiol. Glob. Health.

[11] Stuckelberger, S. et al. SARS-CoV-2 Vaccine Willingness among Pregnant and Breastfeeding Women during the First Pandemic Wave: A Cross-Sectional Study in Switzerland. *Viruses* **13**, 10.3390/v13071199 (2021). 10.3390/v13071199 PMC8310322 34206645

[12] Atyeo C, Alter G (2021). The multifaceted roles of breast milk antibodies. Cell.

[13] Rogier EW (2014). Secretory antibodies in breast milk promote long-term intestinal homeostasis by regulating the gut microbiota and host gene expression. Proc. Natl Acad. Sci. USA.

[14] Sarkar, A., Yoo, J. Y., Valeria Ozorio Dutra, S., Morgan, K. H. & Groer, M. The Association between Early-Life Gut Microbiota and Long-Term Health and Diseases. *J Clin Med* **10**, 10.3390/jcm10030459 (2021). 10.3390/jcm10030459 PMC7865818 33504109

[15] Hooper LV, Littman DR, Macpherson AJ (2012). Interactions between the microbiota and the immune system. Science.

[16] New JS (2020). Neonatal exposure to commensal-bacteria-derived antigens directs polysaccharide-specific B-1 B cell repertoire development. Immunity.

[17] Ng SC (2022). Gut microbiota composition is associated with SARS-CoV-2 vaccine immunogenicity and adverse events. Gut.

[18] Liu F (2022). Longitudinal changes of human milk oligosaccharides, breastmilk microbiome and infant gut microbiome are associated with maternal characteristics. Int. J. Food Sci. Technol..

[19] Murphy K (2017). The Composition of Human Milk and Infant Faecal Microbiota Over the First Three Months of Life: A Pilot Study. Sci. Rep..

[20] Qi C (2022). Lactation-dependent vertical transmission of natural probiotics from the mother to the infant gut through breast milk. Food Funct..

[21] Zhang X (2020). The composition and concordance of lactobacillus populations of infant gut and the corresponding breast-milk and maternal gut. Front. Microbiol..

[22] Łubiech K, Twarużek M (2020). Lactobacillus bacteria in breast milk. Nutrients.

[23] Albrecht M, Arck PC (2020). Vertically transferred immunity in neonates: mothers, mechanisms and mediators. Front. Immunol..

[24] Hung MC, Christodoulides M (2013). The biology of Neisseria adhesins. Biol. (Basel).

[25] Santana-Mederos D (2022). A COVID-19 vaccine candidate composed of the SARS-CoV-2 RBD dimer and Neisseria meningitidis outer membrane vesicles. RSC Chem. Biol..

[26] Hsieh H-S (2022). Gut microbiome profiles and associated metabolic pathways in patients of adult-onset immunodeficiency with anti-interferon-gamma autoantibodies. Sci. Rep..

[27] Roager HM, Licht TR (2018). Microbial tryptophan catabolites in health and disease. Nat. Commun..

[28] Palmieri, O. et al. Microbiome Analysis of Mucosal Ileoanal Pouch in Ulcerative Colitis Patients Revealed Impairment of the Pouches Immunometabolites. *Cells* **10**10.3390/cells10113243(2021). 10.3390/cells10113243 PMC8624401 34831464

[29] Wang Q, Guo A, Sheng M, Zhou H (2021). The changes of respiratory microbiome between mild and severe asthma patients. Microbiol. Immunol..

[30] Hirota, M. et al. Human immune and gut microbial parameters associated with inter-individual variations in COVID-19 mRNA vaccine-induced immunity. *bioRxiv* (2022). 10.1101/2022.08.08.503075. 10.1038/s42003-023-04755-9 PMC10119155 37081096

[31] Bunesova V, Lacroix C, Schwab C (2016). Fucosyllactose and L-fucose utilization of infant Bifidobacterium longum and Bifidobacterium kashiwanohense. BMC Microbiol..

[32] Kononova, S., Litvinova, E., Vakhitov, T., Skalinskaya, M. & Sitkin, S. Acceptive Immunity: The Role of Fucosylated Glycans in Human Host-Microbiome Interactions. *Int J Mol Sci* **22**, 10.3390/ijms22083854(2021). 10.3390/ijms22083854 PMC8068183 33917768

[33] Aakko J (2017). Human milk oligosaccharide categories define the microbiota composition in human colostrum. Benef. Microbes.

[34] Lawson MAE (2020). Breast milk-derived human milk oligosaccharides promote Bifidobacterium interactions within a single ecosystem. ISME J..

[35] Cacho NT, Lawrence RM (2017). Innate Immunity and Breast Milk. Front Immunol..

[36] Choi SSH (2015). Safety evaluation of the human-identical milk monosaccharide, l-fucose. Regulatory Toxicol. Pharmacol..

[37] Nanthakumar NN, Meng D, Newburg DS (2013). Glucocorticoids and microbiota regulate ontogeny of intestinal fucosyltransferase 2 requisite for gut homeostasis. Glycobiology.

[38] Cheng CC (2020). Ecological importance of cross-feeding of the intermediate metabolite 1, 2-propanediol between bacterial gut symbionts. Appl. Environ. Microbiol..

[39] Arpaia N (2013). Metabolites produced by commensal bacteria promote peripheral regulatory T-cell generation. Nature.

[40] Jordan, A., Carding, S. R. & Hall, L. J. The early-life gut microbiome and vaccine efficacy. *The Lancet Microbe* (2022). 10.1016/S2666-5247(22)00185-9. 10.1016/S2666-5247(22)00185-9 36088916

[41] Riskin A (2012). Changes in immunomodulatory constituents of human milk in response to active infection in the nursing infant. Pediatr. Res.

[42] Zimmermann P, Curtis N (2018). The influence of probiotics on vaccine responses – A systematic review. Vaccine.

[43] Zhao T (2020). Influence of gut microbiota on mucosal IgA antibody response to the polio vaccine. NPJ vaccines.

[44] Huda, M. N. et al. Bifidobacterium abundance in early infancy and vaccine response at 2 years of age. *Pediatrics* **143**, 10.1542/peds.2018-1489 (2019). 10.1542/peds.2018-1489 PMC6361348 30674610

[45] Gómez-Torres N (2022). Metataxonomic Analysis of Milk Samples From SARS-CoV-2-Positive and SARS-CoV-2-Negative Women. Front Nutr..

[46] Juárez-Castelán, C. J. et al. The Entero-Mammary Pathway and Perinatal Transmission of Gut Microbiota and SARS-CoV-2. *Int J Mol Sci* **23**, 10.3390/ijms231810306 (2022). 10.3390/ijms231810306 PMC9499685 36142219

[47] Van de Perre P (2003). Transfer of antibody via mother’s milk. Vaccine.

[48] Jakaitis BM, Denning PW (2014). Human breast milk and the gastrointestinal innate immune system. Clin. Perinatol..

[49] Narayanaswamy, V. et al. Neutralizing Antibodies and Cytokines in Breast Milk After Coronavirus Disease 2019 (COVID-19) mRNA Vaccination. *Obstetrics & Gynecology* **139**, 10.1097/AOG.0000000000004661 (2022). 10.1097/AOG.0000000000004661 PMC8759542 35104067

[50] Juncker, H. G. et al. Comparing the human milk antibody response after vaccination with four COVID-19 vaccines: A prospective, longitudinal cohort study in the Netherlands. *eClinicalMedicine* **47**, 10.1016/j.eclinm.2022.101393 (2022). 10.1016/j.eclinm.2022.101393 PMC9013951 35465077

[51] Young BE (2022). Association of Human Milk Antibody Induction, Persistence, and Neutralizing Capacity With SARS-CoV-2 Infection vs mRNA Vaccination. JAMA Pediatrics.

[52] Ricciardi A (2022). Serum and breastmilk SARS-CoV-2 specific antibodies following BNT162b2 vaccine: prolonged protection from SARS-CoV-2 in newborns and older children. Int. J. Infect. Dis..

[53] Yeo, K. T. et al. Neutralizing Activity and SARS-CoV-2 Vaccine mRNA Persistence in Serum and Breastmilk After BNT162b2 Vaccination in Lactating Women. *Frontiers in Immunology* **12** (2022). 10.3389/fimmu.2021.783975 10.3389/fimmu.2021.783975 PMC8787073 35087517

[54] Al-Khafaji AH, Jepsen SD, Christensen KR, Vigsnæs LK (2020). The potential of human milk oligosaccharides to impact the microbiota-gut-brain axis through modulation of the gut microbiota. J. Funct. Foods.

[55] Burki T (2022). Hong Kong’s fifth COVID-19 wave—the worst yet. Lancet Infect. Dis..

[56] Perera, R. A. et al. Serological assays for severe acute respiratory syndrome coronavirus 2 (SARS-CoV-2), March 2020. *Euro Surveill* **25**, 10.2807/1560-7917.Es.2020.25.16.2000421 (2020). 10.2807/1560-7917.ES.2020.25.16.2000421 PMC7189648 32347204

[57] Rio-Aige, K. et al. The Breast Milk Immunoglobulinome. *Nutrients* **13**, 10.3390/nu13061810 (2021). 10.3390/nu13061810 PMC8230140 34073540

[58] Douglas CA (2020). DNA extraction approaches substantially influence the assessment of the human breast milk microbiome. Sci. Rep..

[59] Bolyen E (2019). Reproducible, interactive, scalable and extensible microbiome data science using QIIME 2. Nat. Biotechnol..

[60] Douglas GM (2020). PICRUSt2 for prediction of metagenome functions. Nat. Biotechnol..

