# Supplementary material for: COVID-19 mRNA vaccine-mediated antibodies in human breast milk and their association with breast milk microbiota composition
Source: NPJ Vaccines. 2023 Oct 5;8:151. doi: 10.1038/s41541-023-00745-4 (PMC10556030; doi:10.1038/s41541-023-00745-4)
Supplement: Supplementary file 1 — Supplementary Materials [file 41541_2023_745_MOESM1_ESM.pdf]

Supplemental Information for

COVID-19 mRNA vaccine-mediated antibodies in human breast milk and their association with breast milk microbiota composition

Supplementary Table 1. Association between participant demographics and immunity at different timepoints

Variables	One week after second dose IgA OD value (N=43)	P value	One month after second dose IgA OD value (N=44)	P value
Maternal age	-0.23	0.144	-0.06	0.676
Baby's birth weight (kg)	-0.03	0.833	0.10	0.509
Baby's gestational age	-0.26	0.096	-0.08	0.595
Total number of previous children		0.287		0.657
0	0.16 (0.13, 0.16)		0.10 (0.08, 0.11)	
1	0.20 (0.16, 0.29)		0.11 (0.10, 0.14)	
2	0.25 (0.17, 0.28)		0.13 (0.09, 0.16)	
3	0.18 (0.17, 0.25)		0.12 (0.10, 0.14)	
Delivery type		0.478		0.253
Spontaneous vaginal delivery	0.26 (0.19, 0.28)		0.15 (0.11, 0.16)	
Assisted vaginal Delivery	0.16 (0.15, 0.28)		0.13 (0.10, 0.17)	
Planned C-section	0.19 (0.17, 0.25)		0.10 (0.08, 0.14)	
Emergency C-section	0.17 (0.10, 0.30)		0.11 (0.10, 0.13)	
Labor induced		0.990		0.400
No	0.21 (0.17, 0.28)		0.13 (0.10, 0.16)	
Yes	0.19 (0.15, 0.34)		0.11 (0.08, 0.14)	
Epidural anesthesia used		0.020		0.104
No	0.25 (0.19, 0.28)		0.13 (0.10, 0.16)	
Yes	0.17 (0.12, 0.19)		0.10 (0.08, 0.13)	
Intramuscular analgesia use		0.807		0.349
No	0.20 (0.17, 0.28)		0.12 (0.10, 0.15)	
Yes	0.22 (0.15, 0.27)		0.10 (0.08, 0.14)	
Baby's sex		0.369		0.811
Female	0.21 (0.16, 0.30)		0.13 (0.10, 0.15)	

Supplementary Table 1. Association between participant demographics and immunity at different timepoints

Variables	One week after second dose IgA OD value (N=43)	P value	One month after second dose IgA OD value (N=44)	P value
Male	0.19 (0.16, 0.26)		0.11 (0.09, 0.14)	
Smoked previously		0.375		0.168
No	0.20 (0.16, 0.28)		0.11 (0.09, 0.14)	
Yes	0.28 (0.28, 0.28)		0.17 (0.17, 0.17)	
Partner smokes		0.940		0.796
No	0.20 (0.17, 0.28)		0.11 (0.09, 0.14)	
Yes	0.17 (0.16, 0.30)		0.14 (0.08, 0.16)	
Marital status		0.091		0.844
Married	0.20 (0.16, 0.28)		0.12 (0.09, 0.15)	
Not Married	0.69 (0.69, 0.69)		0.11 (0.11, 0.11)	
Place of birth		0.432		0.729
Hong Kong SAR	0.21 (0.17, 0.28)		0.12 (0.09, 0.15)	
Mainland, China	0.22 (0.14, 0.36)		0.12 (0.11, 0.13)	
Others	0.15 (0.12, 0.20)		0.10 (0.09, 0.14)	
Years living in HK		0.420		0.884
<15 years	0.25 (0.20, 0.28)		0.11 (0.10, 0.16)	
>=15 years	0.19 (0.16, 0.28)		0.12 (0.09, 0.14)	
Education level		0.352		0.673
Postgraduate degree or above	0.21 (0.17, 0.27)		0.11 (0.09, 0.14)	
Under-university level	0.26 (0.18, 0.38)		0.14 (0.10, 0.14)	
University Degree	0.19 (0.16, 0.28)		0.12 (0.08, 0.16)	
Family income		0.297		0.229
HK$40,000 or more	0.19 (0.16, 0.28)		0.11 (0.09, 0.15)	
Under HK$40,000	0.26 (0.25, 0.30)		0.14 (0.11, 0.14)	

Data are correlation coefficients or medians (IQR)

Supplementary Table 2. Factors influencing the baseline breast milk microbiome of the participants (N=44)

	Df	SumOfSqs	R^2	F	Pr(>F)
Total number of previous children	3	8.47E-06	0.06777471	0.96936102	0.488
Lactation stage	40	0.54014	0.94794	1.3656	0.349
Delivery type	3	1.26E-05	0.10097722	1.49758492	0.124
Labor induced	1	4.26E-06	0.03410452	1.48296575	0.19
Intramuscular analgesia use	1	8.08E-06	0.0646456	2.90276642	0.012
Baby's sex	1	2.65E-06	0.02123022	0.91101021	0.475
Smoked previously	1	2.13E-06	0.01707611	0.7296563	0.452
Partner smoke	1	1.05E-06	0.00840764	0.35611484	0.878
Marital Status	1	5.26E-06	0.04211854	1.84676165	0.14
Place of birth	2	5.03E-06	0.04028036	0.86040471	0.579
HK resident	4	1.08E-05	0.08613091	0.91892415	0.516
Education level	5	1.31E-05	0.10445673	0.88646875	0.558
Family Income	3	1.03E-05	0.08235167	1.19656103	0.278

Supplementary Table 3. LEfSe results for each bacterial taxa throughout vaccination regimen

Species	Baseline vs one-week after first dose			Baseline vs one-week after second dose			Baseline vs one-monthafter second dose		
	Enrich group	LDA score	P value	Enrich group	LDA score	P value	Enrich group	LDA score	P value
[Clostridium]_aldenense	NA	NA	NA	NA	NA	NA	NA	NA	NA
Acinetobacter_baumannii	A	4.31690238	0.01850689	C	4.30448163	0.00619704	NA	NA	NA
Acinetobacter_guillouiae	NA	NA	NA	NA	NA	NA	NA	NA	NA
Acinetobacter_radioresistens	B	2.65214107	0.01997803	NA	NA	NA	NA	NA	NA
Acinetobacter_rudis	NA	NA	NA	NA	NA	NA	NA	NA	NA
Acinetobacter_soli	B	1.66153754	0.04446013	C	2.39227209	0.01090213	NA	NA	NA
Acinetobacter_towneri	NA	NA	NA	NA	NA	NA	NA	NA	NA
Acinetobacter_ursingii	B	4.27960427	0.00063271	C	3.28125473	0.00215718	D	4.27497279	0.00828796
Acinetobacter_variabilis	NA	NA	NA	NA	NA	NA	NA	NA	NA
Actinomyces_graevenitzii	NA	NA	NA	NA	NA	NA	NA	NA	NA
Actinomyces_lingnae	NA	NA	NA	NA	NA	NA	NA	NA	NA
Actinomyces_massiliensis	NA	NA	NA	NA	NA	NA	NA	NA	NA
Acutalibacter_muris	NA	NA	NA	NA	NA	NA	NA	NA	NA

Supplementary Table 3. LEfSe results for each bacterial taxa throughout vaccination regimen

Aggregatibacter_actinomycetemcomitans	NA	NA	NA	NA	NA	NA	NA	NA	NA
Aggregatibacter_aphrophilus	NA	NA	NA	NA	NA	NA	NA	NA	NA
Anaerococcus_nagyae	NA	NA	NA	A	2.0934743	0.01079968	NA	NA	NA
Anaerococcus_octavius	NA	NA	NA	A	1.70752564	0.00675453	A	1.7175902	0.02336219
Anaerococcus_vaginalis	NA	NA	NA	NA	NA	NA	NA	NA	NA
Aquabacterium_citratiphilum	NA	NA	NA	NA	NA	NA	A	1.54656048	0.02213389
Arcomarinus_aquaticus	NA	NA	NA	NA	NA	NA	NA	NA	NA
Arthrobacter_russicus	NA	NA	NA	A	1.94919302	0.0442347	A	1.78402183	0.04187977
Bacillus_thermoamylovorans	B	1.63971221	0.04187977	NA	NA	NA	NA	NA	NA
bacterium_Desulfovibrio	NA	NA	NA	NA	NA	NA	NA	NA	NA
Bacteroides_caecimuris	NA	NA	NA	A	2.03203974	6.49E-05	A	2.0732493	5.42E-05
Bacteroides_coprocola	NA	NA	NA	NA	NA	NA	A	1.62398051	0.02213389
Bacteroides_dorei	NA	NA	NA	NA	NA	NA	NA	NA	NA

Supplementary Table 3. LEfSe results for each bacterial taxa throughout vaccination regimen

Bacteroides_fragilis	NA	NA	NA	NA	NA	NA	A	1.67859626	0.02213389
Bacteroides_plebeius	NA	NA	NA	A	1.94035678	0.00675453	NA	NA	NA
Bacteroides_sartorii	NA	NA	NA	NA	NA	NA	NA	NA	NA
Bacteroides_stercoris	NA	NA	NA	NA	NA	NA	NA	NA	NA
Bacteroides_vulgatus	NA	NA	NA	A	1.91265337	0.03285333	NA	NA	NA
Bifidobacterium_bifidum	NA	NA	NA	NA	NA	NA	A	1.86206851	0.03023065
Bifidobacterium_breve	NA	NA	NA	NA	NA	NA	NA	NA	NA
Bifidobacterium_dentium	NA	NA	NA	NA	NA	NA	NA	NA	NA
Bifidobacterium_longum	NA	NA	NA	NA	NA	NA	NA	NA	NA
Brevundimonas_olei	NA	NA	NA	C	3.13528752	0.0455406	NA	NA	NA
Brevundimonas_staleyi	NA	NA	NA	NA	NA	NA	NA	NA	NA
Calothrix_sp.	NA	NA	NA	NA	NA	NA	NA	NA	NA
Campylobacter_concisus	NA	NA	NA	NA	NA	NA	NA	NA	NA
Campylobacter_showae	NA	NA	NA	NA	NA	NA	D	1.83162313	0.04836398
Capnocytophaga_gingivalis	NA	NA	NA	NA	NA	NA	NA	NA	NA

Supplementary Table 3. LEfSe results for each bacterial taxa throughout vaccination regimen

Capnocytophaga_leadbetteri	NA	NA	NA	NA	NA	NA	NA	NA	NA
Capnocytophaga_sputigena	NA	NA	NA	NA	NA	NA	NA	NA	NA
Castellaniella_defragrans	NA	NA	NA	NA	NA	NA	NA	NA	NA
Chryseobacterium_hominis	NA	NA	NA	C	2.14529463	0.02742341	D	2.25859826	0.00465768
Chryseobacterium_piscium	NA	NA	NA	NA	NA	NA	NA	NA	NA
Chryseobacterium_stagni	NA	NA	NA	NA	NA	NA	NA	NA	NA
Chryseobacterium_taklimakanense	NA	NA	NA	NA	NA	NA	NA	NA	NA
Clostridiaceae_bacterium	NA	NA	NA	A	2.06220828	0.00664691	A	2.14395412	0.00011022
Clostridiales_bacterium_Clostridia_UCG-014	NA	NA	NA	C	2.99662775	0.01126005	D	2.01673941	0.01084316
Clostridium_sp._Incertae_Sedis	NA	NA	NA	NA	NA	NA	NA	NA	NA
Clostridium_sp._un_Oscillospiraceae	NA	NA	NA	A	1.71675156	0.00358085	A	1.78428461	0.0032276
Collinsella_tanakaei	NA	NA	NA	NA	NA	NA	NA	NA	NA
Comamonas_aquatica	NA	NA	NA	NA	NA	NA	A	1.73784148	0.00167675
Comamonas_denitrificans	NA	NA	NA	NA	NA	NA	NA	NA	NA
Corynebacterium_amycolatum	NA	NA	NA	NA	NA	NA	NA	NA	NA
Corynebacterium_appendicis	NA	NA	NA	NA	NA	NA	NA	NA	NA

Supplementary Table 3. LEfSe results for each bacterial taxa throughout vaccination regimen

Corynebacterium_bovis	NA	NA	NA	NA	NA	NA	NA	NA	NA
Corynebacterium_durum	NA	NA	NA	NA	NA	NA	NA	NA	NA
Corynebacterium_jeikeium	NA	NA	NA	NA	NA	NA	NA	NA	NA
Corynebacterium_kroppenstedtii	NA	NA	NA	NA	NA	NA	NA	NA	NA
Corynebacterium_lipophiloflavum	NA	NA	NA	NA	NA	NA	NA	NA	NA
Corynebacterium_mycetoides	NA	NA	NA	NA	NA	NA	NA	NA	NA
Corynebacterium_propinquum	NA	NA	NA	NA	NA	NA	NA	NA	NA
Corynebacterium_suicordis	NA	NA	NA	NA	NA	NA	NA	NA	NA
Corynebacterium_urealyticum	NA	NA	NA	NA	NA	NA	NA	NA	NA
Cutibacterium_avidum	NA	NA	NA	NA	NA	NA	NA	NA	NA
Cutibacterium_granulosum	NA	NA	NA	A	2.97784251	0.027992	NA	NA	NA
Cytophaga_hutchinsonii	NA	NA	NA	NA	NA	NA	NA	NA	NA
Deinococcus_antarcticus	NA	NA	NA	NA	NA	NA	NA	NA	NA
Deinococcus_radiodurans	NA	NA	NA	NA	NA	NA	NA	NA	NA
Deinococcus_sp.	NA	NA	NA	NA	NA	NA	NA	NA	NA
Deinococcus_wulumuqiensis	NA	NA	NA	NA	NA	NA	NA	NA	NA
Dorea_formicigenerans	NA	NA	NA	NA	NA	NA	NA	NA	NA
Dubosiella_newyorkensis	NA	NA	NA	NA	NA	NA	D	2.01946277	0.04187977

Supplementary Table 3. LEfSe results for each bacterial taxa throughout vaccination regimen

Empedobacter_brevis	B	2.80808006	0.01585632	NA	NA	NA	NA	NA	NA
Endobacter_medicaginis	NA	NA	NA	NA	NA	NA	NA	NA	NA
Enterococcus_cecorum	NA	NA	NA	NA	NA	NA	A	1.72597021	0.01170128
Eubacterium_sp._un_Lachnospiraceae	NA	NA	NA	NA	NA	NA	NA	NA	NA
Facklamia_hominis	NA	NA	NA	NA	NA	NA	NA	NA	NA
Flavobacterium_qiangtangense	NA	NA	NA	NA	NA	NA	NA	NA	NA
Fusobacterium_periodonticum	NA	NA	NA	NA	NA	NA	NA	NA	NA
Fusobacterium_sp.	NA	NA	NA	NA	NA	NA	NA	NA	NA
Gracilibacteria_bacterium_Gracilibacteria	NA	NA	NA	NA	NA	NA	NA	NA	NA
Gracilibacteria_bacterium_JGI_0000069-P22	NA	NA	NA	NA	NA	NA	NA	NA	NA
Haemophilus_haemolyticus	B	2.49015168	0.00448494	NA	NA	NA	NA	NA	NA
Haemophilus_parahaemolyticus	B	2.12978337	0.02213389	NA	NA	NA	NA	NA	NA
Haemophilus_sp.	NA	NA	NA	NA	NA	NA	NA	NA	NA
Haemophilus_sputorum	NA	NA	NA	NA	NA	NA	NA	NA	NA
Helicobacter_rodentium	A	2.37253329	0.00924467	A	2.45152656	7.37E-06	A	2.51045731	5.91E-06

Supplementary Table 3. LEfSe results for each bacterial taxa throughout vaccination regimen

human_gut_[Eubacterium]_coprostanoligenes_group	NA	NA	NA	NA	NA	NA	NA	NA	NA
Idiomarina_sp.	NA	NA	NA	NA	NA	NA	NA	NA	NA
Ignatzschineria_sp.	NA	NA	NA	NA	NA	NA	NA	NA	NA
Inostemma_sp.	NA	NA	NA	NA	NA	NA	NA	NA	NA
Kocuria_marina	NA	NA	NA	NA	NA	NA	NA	NA	NA
Kocuria_palustris	NA	NA	NA	NA	NA	NA	NA	NA	NA
Koukoulia_aurantiaca	NA	NA	NA	NA	NA	NA	NA	NA	NA
Lactobacillus_aviarius	NA	NA	NA	A	3.40520331	0.01452433	A	3.36226108	0.00131456
Lactobacillus_fermentum	NA	NA	NA	NA	NA	NA	NA	NA	NA
Lactobacillus_gasseri	NA	NA	NA	NA	NA	NA	NA	NA	NA
Lactobacillus_iners	NA	NA	NA	NA	NA	NA	NA	NA	NA
Lactobacillus_intestinalis	A	1.69656573	0.02213389	NA	NA	NA	NA	NA	NA
Leptotrichia_buccalis	NA	NA	NA	NA	NA	NA	NA	NA	NA
Leptotrichia_hongkongensis	NA	NA	NA	NA	NA	NA	NA	NA	NA
Leptotrichia_shahii	NA	NA	NA	NA	NA	NA	NA	NA	NA
Leptotrichia_sp.	NA	NA	NA	NA	NA	NA	A	1.63785887	0.02213389
Leuconostoc_carnosum	NA	NA	NA	NA	NA	NA	NA	NA	NA

Supplementary Table 3. LEfSe results for each bacterial taxa throughout vaccination regimen

Lysinibacillus_alkalisoli	NA	NA	NA	NA	NA	NA	NA	NA	NA
Marinobacterium_marisflavi	NA	NA	NA	NA	NA	NA	D	1.70924177	0.02213389
Megasphaera_micronuciformis	NA	NA	NA	NA	NA	NA	NA	NA	NA
Moraxella_atlantae	NA	NA	NA	C	2.96230507	0.00187372	D	3.22273387	0.01142775
Moraxella_porci	NA	NA	NA	C	2.0439466	0.00557638	NA	NA	NA
Mucilaginibacter_oryzae	NA	NA	NA	NA	NA	NA	NA	NA	NA
Mucispirillum_sp.	NA	NA	NA	NA	NA	NA	NA	NA	NA
Myroides_odoratus	NA	NA	NA	NA	NA	NA	NA	NA	NA
Neisseria_elongata	NA	NA	NA	NA	NA	NA	D	2.17580805	0.02411406
Neisseria_oralis	NA	NA	NA	NA	NA	NA	NA	NA	NA
Neisseria_perflava	NA	NA	NA	NA	NA	NA	NA	NA	NA
Ottowia_sp.	NA	NA	NA	NA	NA	NA	NA	NA	NA
Paenibacillus_macerans	NA	NA	NA	C	1.69394297	0.04288962	NA	NA	NA
Parabacteroides_merdae	NA	NA	NA	NA	NA	NA	NA	NA	NA
Paracraurococcus_sp.	NA	NA	NA	NA	NA	NA	NA	NA	NA
Parapusillimonas_granuli	NA	NA	NA	NA	NA	NA	NA	NA	NA

Supplementary Table 3. LEfSe results for each bacterial taxa throughout vaccination regimen

Pararheinheimera_chironomi	NA	NA	NA	NA	NA	NA	NA	NA	NA
Parasegetibacter_luojiensis	NA	NA	NA	NA	NA	NA	NA	NA	NA
Patulibacter_sp.	NA	NA	NA	NA	NA	NA	NA	NA	NA
Peredibacter_starrii	NA	NA	NA	C	1.693103	0.03952802	NA	NA	NA
Piscicoccus_intestinalis	NA	NA	NA	NA	NA	NA	NA	NA	NA
Polyangium_sp.	NA	NA	NA	NA	NA	NA	NA	NA	NA
Porphyromonas_pasteri	NA	NA	NA	NA	NA	NA	NA	NA	NA
Prevotella_aurantiaca	NA	NA	NA	NA	NA	NA	NA	NA	NA
Prevotella_copri	NA	NA	NA	NA	NA	NA	NA	NA	NA
Prevotella_histicola	NA	NA	NA	NA	NA	NA	D	3.17705837	0.01033648
Prevotella_jejuni	NA	NA	NA	NA	NA	NA	NA	NA	NA
Prevotella_loescheii	NA	NA	NA	NA	NA	NA	NA	NA	NA
Prevotella_melaninogenica	NA	NA	NA	C	4.1285659	0.03468801	D	3.83703589	0.04182696
Prevotella_nanceiensis	NA	NA	NA	NA	NA	NA	NA	NA	NA
Prevotella_pallens	NA	NA	NA	NA	NA	NA	NA	NA	NA
Prevotella_salivae	NA	NA	NA	NA	NA	NA	NA	NA	NA
Pseudomonas_balearica	NA	NA	NA	NA	NA	NA	NA	NA	NA
Pseudomonas_guguanensis	NA	NA	NA	NA	NA	NA	NA	NA	NA

Supplementary Table 3. LEfSe results for each bacterial taxa throughout vaccination regimen

Pseudomonas_luteola	NA	NA	NA	NA	NA	NA	NA	NA	NA
Pseudoxanthomonas_kaohsiungensis	NA	NA	NA	A	1.54189822	0.02366549	A	1.55790232	0.02213389
Rhodococcus_corynebacterioides	NA	NA	NA	NA	NA	NA	NA	NA	NA
Rivicola_pingtungensis	NA	NA	NA	NA	NA	NA	NA	NA	NA
Roseomonas_aestuarii	NA	NA	NA	NA	NA	NA	D	3.08348454	0.00086305
Roseomonas_gilardii	B	1.83127452	0.0314702	C	2.21938428	0.01620112	D	2.75426559	0.00175211
Rothia_aeria	NA	NA	NA	NA	NA	NA	NA	NA	NA
Rothia_kristinae	NA	NA	NA	NA	NA	NA	NA	NA	NA
Rothia_mucilaginosa	NA	NA	NA	NA	NA	NA	NA	NA	NA
Rothia_sp.	NA	NA	NA	A	1.90158329	0.02366549	A	1.85221663	0.02213389
rumen_bacterium_Coprococcus	NA	NA	NA	NA	NA	NA	NA	NA	NA
Ruminococcus_bicirculans	NA	NA	NA	NA	NA	NA	NA	NA	NA
Ruminococcus_callidus	NA	NA	NA	NA	NA	NA	NA	NA	NA
Ruminococcus_flavefaciens	NA	NA	NA	NA	NA	NA	A	1.93532706	0.04187977
Saccharomonospora_viridis	NA	NA	NA	NA	NA	NA	NA	NA	NA
Schaalia_odontolytica	NA	NA	NA	NA	NA	NA	NA	NA	NA

Supplementary Table 3. LEfSe results for each bacterial taxa throughout vaccination regimen

Selenomonas_sp.	NA	NA	NA	NA	NA	NA	NA	NA	NA
Siphonobacter_sp.	NA	NA	NA	NA	NA	NA	NA	NA	NA
Sphingomonas_panni	NA	NA	NA	NA	NA	NA	NA	NA	NA
Sphingomonas_phyllosphaerae	NA	NA	NA	NA	NA	NA	NA	NA	NA
SR1_bacterium	NA	NA	NA	NA	NA	NA	NA	NA	NA
Staphylococcus_equorum	NA	NA	NA	NA	NA	NA	NA	NA	NA
Stenotrophomonas_acidaminiphila	NA	NA	NA	NA	NA	NA	D	2.59801176	0.02904233
Stenotrophomonas_rhizophila	NA	NA	NA	NA	NA	NA	NA	NA	NA
Streptococcus_anginosus	NA	NA	NA	NA	NA	NA	NA	NA	NA
Streptococcus_parasuis	NA	NA	NA	NA	NA	NA	NA	NA	NA
Streptococcus_peroris	NA	NA	NA	NA	NA	NA	NA	NA	NA
Streptococcus_salivarius	NA	NA	NA	NA	NA	NA	NA	NA	NA
Thermus_thermophilus	NA	NA	NA	NA	NA	NA	NA	NA	NA
TM7_phylum	NA	NA	NA	NA	NA	NA	NA	NA	NA
un_[Eubacterium]_coprostanoligenes_group	NA	NA	NA	A	2.51848195	0.00917398	A	2.64461681	4.58E-06
un_[Eubacterium]_hallii_group	NA	NA	NA	NA	NA	NA	A	2.16486553	0.01492197
un_[Eubacterium]_oxidoreducens_group	NA	NA	NA	NA	NA	NA	NA	NA	NA

Supplementary Table 3. LEfSe results for each bacterial taxa throughout vaccination regimen

un_[Eubacterium]_ruminantium_group	NA	NA	NA	NA	NA	NA	A	2.44947113	0.04187977
un_[Eubacterium]_ventriosum_group	B	1.69635165	0.04187977	C	1.52403928	0.01074057	NA	NA	NA
un_[Eubacterium]_xylanophilum_group	NA	NA	NA	NA	NA	NA	NA	NA	NA
un_[Ruminococcus]_gauvreauii_group	NA	NA	NA	NA	NA	NA	NA	NA	NA
un_[Ruminococcus]_gnavus_group	NA	NA	NA	NA	NA	NA	A	2.15261313	0.026261
un_[Ruminococcus]_torques_group	NA	NA	NA	NA	NA	NA	NA	NA	NA
un_A0839	NA	NA	NA	A	1.51744257	0.02366549	A	1.58066899	0.02213389
un_Abiotrophia	NA	NA	NA	NA	NA	NA	NA	NA	NA
un_Acetoanaerobium	NA	NA	NA	NA	NA	NA	NA	NA	NA
un_Acholeplasma	NA	NA	NA	NA	NA	NA	NA	NA	NA
un_Acidaminococcus	NA	NA	NA	NA	NA	NA	NA	NA	NA
un_Acidovorax	NA	NA	NA	NA	NA	NA	D	2.52361577	0.0032276
un_Acinetobacter	NA	NA	NA	C	3.91957258	0.00304027	NA	NA	NA
un_Actinobacillus	B	1.70083939	0.0103969	C	2.08798222	0.04492668	NA	NA	NA

Supplementary Table 3. LEfSe results for each bacterial taxa throughout vaccination regimen

un_Actinomadura	NA	NA	NA	NA	NA	NA	NA	NA	NA
un_Actinomyces	NA	NA	NA	NA	NA	NA	NA	NA	NA
un_AEGEAN-169_marine_group	NA	NA	NA	NA	NA	NA	NA	NA	NA
un_Aerococcus	NA	NA	NA	NA	NA	NA	A	2.35328825	0.00183558
un_Aeromicrobium	NA	NA	NA	NA	NA	NA	D	1.68232456	0.02213389
un_Aeromonas	NA	NA	NA	NA	NA	NA	NA	NA	NA
un_Agathobacter	NA	NA	NA	NA	NA	NA	NA	NA	NA
un_Aggregatibacter	NA	NA	NA	NA	NA	NA	NA	NA	NA
un_Akkermansia	NA	NA	NA	A	3.63458755	1.12E-06	A	3.72706226	2.58E-05
un_Alcaligenaceae	NA	NA	NA	NA	NA	NA	NA	NA	NA
un_Alcaligenes	NA	NA	NA	A	1.74926164	0.00188365	A	1.84811541	0.026261
un_Alishewanella	NA	NA	NA	NA	NA	NA	NA	NA	NA
un_Alistipes	NA	NA	NA	NA	NA	NA	A	1.82735732	0.00047764
un_Alkanindiges	NA	NA	NA	NA	NA	NA	NA	NA	NA
un_Allobaculum	A	1.76575297	0.02208295	A	1.9391714	0.0001303	A	1.94941683	0.00011022

Supplementary Table 3. LEfSe results for each bacterial taxa throughout vaccination regimen

un_Alloprevotella	NA	NA	NA	NA	NA	NA	NA	NA	NA
un_Allorhizobium-Neorhizobium-Pararhizobium-Rhizobium	B	2.98677001	0.02983076	NA	NA	NA	D	3.7884748	0.04490721
un_Alteromonadaceae	NA	NA	NA	NA	NA	NA	NA	NA	NA
un_Alysiella	NA	NA	NA	NA	NA	NA	NA	NA	NA
un_Amaricoccus	NA	NA	NA	NA	NA	NA	NA	NA	NA
un_Anaerococcus	NA	NA	NA	NA	NA	NA	A	1.58586622	0.01170128
un_Anaerostipes	NA	NA	NA	NA	NA	NA	NA	NA	NA
un_Anoxybacillus	B	2.09082907	0.00044459	NA	NA	NA	NA	NA	NA
un_Aquabacterium	NA	NA	NA	NA	NA	NA	NA	NA	NA
un_Arcobacter	NA	NA	NA	NA	NA	NA	NA	NA	NA
un_Arsenicicoccus	NA	NA	NA	NA	NA	NA	NA	NA	NA
un_ASF356	NA	NA	NA	NA	NA	NA	NA	NA	NA
un_Asticcacaulis	NA	NA	NA	NA	NA	NA	NA	NA	NA
un_Atopobium	NA	NA	NA	NA	NA	NA	D	2.34595009	0.00574396
un_Atopostipes	NA	NA	NA	NA	NA	NA	NA	NA	NA
un_Aureimonas	NA	NA	NA	NA	NA	NA	NA	NA	NA
un_Auricoccus-Abyssicoccus	NA	NA	NA	NA	NA	NA	NA	NA	NA

Supplementary Table 3. LEfSe results for each bacterial taxa throughout vaccination regimen

un_Azorhizobium	NA	NA	NA	NA	NA	NA	NA	NA	NA
un_Bacilli	A	1.96601304	0.0098417	A	1.96407979	0.00129704	NA	NA	NA
un_Bacillus	B	3.9729011	0.00440434	NA	NA	NA	NA	NA	NA
un_Bacteria	NA	NA	NA	NA	NA	NA	NA	NA	NA
un_Bacteroidales	NA	NA	NA	NA	NA	NA	NA	NA	NA
un_Bacteroides	NA	NA	NA	A	2.85379794	0.00049301	A	2.79094275	0.00120902
un_Barnesiella	NA	NA	NA	NA	NA	NA	NA	NA	NA
un_Bdellovibrio	NA	NA	NA	NA	NA	NA	A	1.61974895	0.02213389
un_Beijerinckiaceae	NA	NA	NA	NA	NA	NA	NA	NA	NA
un_Bergeyella	B	3.07119487	0.03497732	NA	NA	NA	NA	NA	NA
un_Bifidobacterium	NA	NA	NA	NA	NA	NA	NA	NA	NA
un_Bilophila	NA	NA	NA	NA	NA	NA	NA	NA	NA
un_Blastococcus	NA	NA	NA	NA	NA	NA	NA	NA	NA
un_Blautia	NA	NA	NA	NA	NA	NA	NA	NA	NA
un_Bordetella	NA	NA	NA	NA	NA	NA	NA	NA	NA
un_Bosea	NA	NA	NA	NA	NA	NA	NA	NA	NA

Supplementary Table 3. LEfSe results for each bacterial taxa throughout vaccination regimen

un_Brachybacterium	NA	NA	NA	NA	NA	NA	NA	NA	NA
un_Bradyrhizobium	NA	NA	NA	NA	NA	NA	NA	NA	NA
un_Brevibacillus	NA	NA	NA	NA	NA	NA	NA	NA	NA
un_Brevibacterium	NA	NA	NA	NA	NA	NA	NA	NA	NA
un_Brevundimonas	B	3.02944127	0.0403443	C	2.39918908	0.0249322	D	3.24302977	0.03025001
un_Burkholderia-Caballeronia-Paraburkholderia	NA	NA	NA	C	1.53836006	0.00157719	NA	NA	NA
un_Butyricicoccus	NA	NA	NA	NA	NA	NA	NA	NA	NA
un_CAG-352	NA	NA	NA	NA	NA	NA	NA	NA	NA
un_CAG-56	NA	NA	NA	NA	NA	NA	NA	NA	NA
un_Candidatus_Alysiosphaera	NA	NA	NA	NA	NA	NA	NA	NA	NA
un_Candidatus_Saccharimonas	NA	NA	NA	NA	NA	NA	NA	NA	NA
un_Capnocytophaga	NA	NA	NA	NA	NA	NA	NA	NA	NA
un_Cardiobacterium	NA	NA	NA	NA	NA	NA	NA	NA	NA
un_Castellaniella	NA	NA	NA	A	3.52859149	0.04751962	A	3.55770336	0.00540806
un_Catenibacterium	NA	NA	NA	NA	NA	NA	NA	NA	NA
un_Catonella	NA	NA	NA	NA	NA	NA	NA	NA	NA
un_Caulobacter	NA	NA	NA	NA	NA	NA	NA	NA	NA
un_CHAB-XI-27	NA	NA	NA	NA	NA	NA	NA	NA	NA

Supplementary Table 3. LEfSe results for each bacterial taxa throughout vaccination regimen

un_Chitinophagaceae	NA	NA	NA	C	2.07277684	0.02651745	NA	NA	NA
un_CHKCI001	B	2.12370019	0.02897775	NA	NA	NA	NA	NA	NA
un_Chloroplast	NA	NA	NA	NA	NA	NA	D	4.33035019	0.00885572
un_Christensenellaceae_R-7_group	NA	NA	NA	NA	NA	NA	NA	NA	NA
un_Chryseobacterium	NA	NA	NA	NA	NA	NA	NA	NA	NA
un_Citrobacter	NA	NA	NA	NA	NA	NA	NA	NA	NA
un_Cloacibacterium	NA	NA	NA	NA	NA	NA	NA	NA	NA
un_Clostridia_UCG-014	NA	NA	NA	NA	NA	NA	NA	NA	NA
un_Clostridiaceae	NA	NA	NA	NA	NA	NA	NA	NA	NA
un_Clostridium_sensu_stricto_1	NA	NA	NA	A	3.12964895	0.04352989	A	3.21400457	0.02296304
un_Colidextribacter	NA	NA	NA	A	1.75559502	0.01079968	NA	NA	NA
un_Collinsella	NA	NA	NA	NA	NA	NA	NA	NA	NA
un_Comamonadaceae	A	3.24621527	0.02031733	NA	NA	NA	NA	NA	NA
un_Comamonas	NA	NA	NA	NA	NA	NA	NA	NA	NA
un_Coprococcus	NA	NA	NA	NA	NA	NA	NA	NA	NA

Supplementary Table 3. LEfSe results for each bacterial taxa throughout vaccination regimen

un_Coriobacteriaceae_UCG-002	NA	NA	NA	A	1.60386737	0.00050657	A	1.57275594	0.00043966
un_Corynebacteriaceae	NA	NA	NA	NA	NA	NA	D	3.00370434	0.00154975
un_Corynebacterium	NA	NA	NA	NA	NA	NA	NA	NA	NA
un_Cutibacterium	NA	NA	NA	NA	NA	NA	NA	NA	NA
un_Dermacoccaceae	NA	NA	NA	NA	NA	NA	D	2.92013537	0.00078141
un_Dermacoccus	NA	NA	NA	NA	NA	NA	NA	NA	NA
un_Desulfovibrio	NA	NA	NA	NA	NA	NA	NA	NA	NA
un_Desulfovibrionaceae	NA	NA	NA	A	2.66137285	3.47E-06	A	2.72758776	2.74E-06
un_Devosia	NA	NA	NA	NA	NA	NA	NA	NA	NA
un_Dialister	NA	NA	NA	NA	NA	NA	A	1.73455432	0.04898131
un_Diaphorobacter	NA	NA	NA	NA	NA	NA	NA	NA	NA
un_Dietzia	NA	NA	NA	NA	NA	NA	NA	NA	NA
un_Dolosigranulum	NA	NA	NA	NA	NA	NA	NA	NA	NA
un_Dorea	NA	NA	NA	NA	NA	NA	NA	NA	NA
un_Dubosiella	NA	NA	NA	A	1.94231869	0.00050657	A	1.9536549	0.00043966

Supplementary Table 3. LEfSe results for each bacterial taxa throughout vaccination regimen

un_Duganella	B	2.23965261	0.00786483	NA	NA	NA	NA	NA	NA
un_Edaphobaculum	NA	NA	NA	NA	NA	NA	NA	NA	NA
un_Eggerthella	NA	NA	NA	NA	NA	NA	NA	NA	NA
un_Eikenella	NA	NA	NA	NA	NA	NA	NA	NA	NA
un_Elev-16S-573	NA	NA	NA	NA	NA	NA	NA	NA	NA
un_Elstera	NA	NA	NA	NA	NA	NA	NA	NA	NA
un_Empedobacter	B	1.96195675	0.01452762	NA	NA	NA	NA	NA	NA
un_Enhydrobacter	B	3.73471677	0.03333164	NA	NA	NA	NA	NA	NA
un_Enterobacter	NA	NA	NA	NA	NA	NA	NA	NA	NA
un_Enterobacterales	NA	NA	NA	NA	NA	NA	NA	NA	NA
un_Enterobacteriaceae	NA	NA	NA	NA	NA	NA	A	4.20625746	0.04006881
un_Enterococcus	NA	NA	NA	NA	NA	NA	A	4.11529669	0.0327081
un_Erwiniaceae	NA	NA	NA	NA	NA	NA	NA	NA	NA
un_Erysipelotrichaceae_UCG-003	NA	NA	NA	NA	NA	NA	NA	NA	NA
un_Escherichia-Shigella	NA	NA	NA	NA	NA	NA	NA	NA	NA

Supplementary Table 3. LEfSe results for each bacterial taxa throughout vaccination regimen

un_Exiguobacterium	NA	NA	NA	NA	NA	NA	A	1.61423999	0.02213389
un_F0332	NA	NA	NA	NA	NA	NA	NA	NA	NA
un_F082	NA	NA	NA	NA	NA	NA	NA	NA	NA
un_Faecalibacterium	NA	NA	NA	NA	NA	NA	NA	NA	NA
un_Faecalibaculum	NA	NA	NA	A	2.033305	0.00025846	A	2.07793474	0.00022146
un_Faecalitalea	NA	NA	NA	NA	NA	NA	A	1.7846408	0.00616415
un_Finegoldia	NA	NA	NA	NA	NA	NA	NA	NA	NA
un_Firmicutes	NA	NA	NA	NA	NA	NA	NA	NA	NA
un_Flaviflexus	NA	NA	NA	NA	NA	NA	A	2.87053716	0.02691874
un_Flavobacterium	B	2.47878683	0.02548366	NA	NA	NA	NA	NA	NA
un_Flavonifractor	NA	NA	NA	NA	NA	NA	NA	NA	NA
un_Fluviicola	NA	NA	NA	NA	NA	NA	NA	NA	NA
un_Formosa	NA	NA	NA	NA	NA	NA	D	1.97042436	0.02213389
un_Friedmanniella	NA	NA	NA	NA	NA	NA	NA	NA	NA

Supplementary Table 3. LEfSe results for each bacterial taxa throughout vaccination regimen

un_Fusicatenibacter	A	2.25101786	0.03517015	NA	NA	NA	NA	NA	NA
un_Fusobacterium	NA	NA	NA	NA	NA	NA	NA	NA	NA
un_Gaiellales	NA	NA	NA	NA	NA	NA	NA	NA	NA
un_Gemella	NA	NA	NA	NA	NA	NA	NA	NA	NA
un_Gemmobacter	NA	NA	NA	C	1.94657187	0.03952802	NA	NA	NA
un_Geodermatophilus	NA	NA	NA	NA	NA	NA	NA	NA	NA
un_Gordonia	B	2.72176461	0.00050575	C	2.11542012	0.01853374	D	2.41188291	0.02279654
un_Granulicatella	NA	NA	NA	NA	NA	NA	NA	NA	NA
un_Haematobacter	NA	NA	NA	NA	NA	NA	NA	NA	NA
un_Haemophilus	NA	NA	NA	NA	NA	NA	NA	NA	NA
un_Halarcobacter	NA	NA	NA	NA	NA	NA	NA	NA	NA
un_Haliangium	NA	NA	NA	A	1.58513104	0.02366549	A	1.69192122	0.02213389
un_Halomonadaceae	NA	NA	NA	A	2.35217142	0.00199481	A	2.33157628	5.91E-06
un_Halomonas	NA	NA	NA	A	2.33846542	3.19E-05	A	2.35881299	2.63E-05
un_Helcococcus	NA	NA	NA	NA	NA	NA	NA	NA	NA

Supplementary Table 3. LEfSe results for each bacterial taxa throughout vaccination regimen

un_Helicobacter	NA	NA	NA	NA	NA	NA	NA	NA	NA
un_Herbaspirillum	NA	NA	NA	NA	NA	NA	NA	NA	NA
un_Holdemanella	NA	NA	NA	NA	NA	NA	A	1.67340563	0.02213389
un_Incertae_Sedis_Incertae_Sedis_Ruminococcaceae	NA	NA	NA	NA	NA	NA	NA	NA	NA
un_Intrasporangiaceae	NA	NA	NA	NA	NA	NA	NA	NA	NA
un_Isoptericola	NA	NA	NA	NA	NA	NA	NA	NA	NA
un_Janthinobacterium	B	2.89433899	0.02258218	NA	NA	NA	NA	NA	NA
un_Jeotgalicoccus	NA	NA	NA	NA	NA	NA	NA	NA	NA
un_Johnsonella	NA	NA	NA	NA	NA	NA	NA	NA	NA
un_Kingella	NA	NA	NA	NA	NA	NA	NA	NA	NA
un_Klebsiella	NA	NA	NA	C	4.34168793	0.00275972	A	4.20903989	0.01918015
un_Kocuria	NA	NA	NA	NA	NA	NA	NA	NA	NA
un_Kurthia	NA	NA	NA	A	1.52204076	0.00675453	NA	NA	NA
un_Kytococcus	NA	NA	NA	NA	NA	NA	NA	NA	NA
un_Lachnoanaerobaculum	NA	NA	NA	NA	NA	NA	D	2.23098276	0.0131535

Supplementary Table 3. LEfSe results for each bacterial taxa throughout vaccination regimen

un_Lachnoclostridium	NA	NA	NA	NA	NA	NA	A	2.22537524	0.00235948
un_Lachnospiraceae	NA	NA	NA	A	2.60817818	0.02580041	A	2.5697883	0.02146579
un_Lachnospiraceae_NC2004_group	NA	NA	NA	NA	NA	NA	NA	NA	NA
un_Lachnospiraceae_ND3007_group	NA	NA	NA	NA	NA	NA	NA	NA	NA
un_Lachnospiraceae_NK4A136_group	NA	NA	NA	A	2.36648131	0.01184528	A	2.37374337	0.01260039
un_Lachnospiraceae_UCG-001	NA	NA	NA	A	1.62685386	0.00050657	A	1.71168858	0.00043966
un_Lachnospiraceae_UCG-004	NA	NA	NA	NA	NA	NA	NA	NA	NA
un_Lachnospiraceae_UCG-006	NA	NA	NA	NA	NA	NA	NA	NA	NA
un_Lachnospiraceae_UCG-010	NA	NA	NA	NA	NA	NA	NA	NA	NA
un_Lactobacillales	NA	NA	NA	NA	NA	NA	NA	NA	NA
un_Lactobacillus	NA	NA	NA	A	3.24773106	0.01875368	NA	NA	NA
un_Lactococcus	NA	NA	NA	NA	NA	NA	NA	NA	NA
un_Lautropia	NA	NA	NA	NA	NA	NA	A	3.75162106	0.0303737
un_Lawsonella	NA	NA	NA	NA	NA	NA	NA	NA	NA

Supplementary Table 3. LEfSe results for each bacterial taxa throughout vaccination regimen

un_Leptotrichia	NA	NA	NA	C	3.65952951	0.02645427	NA	NA	NA
un_Leucobacter	NA	NA	NA	NA	NA	NA	NA	NA	NA
un_Macellibacteroides	NA	NA	NA	NA	NA	NA	NA	NA	NA
un_Macrococcus	NA	NA	NA	NA	NA	NA	NA	NA	NA
un_Marine_Methylotrophic_Group_2	NA	NA	NA	NA	NA	NA	NA	NA	NA
un_Marmoricola	NA	NA	NA	NA	NA	NA	NA	NA	NA
un_Massilia	NA	NA	NA	NA	NA	NA	NA	NA	NA
un_Megamonas	NA	NA	NA	C	1.67601601	0.02126613	NA	NA	NA
un_Methylobacterium-Methylorubrum	NA	NA	NA	NA	NA	NA	NA	NA	NA
un_Micavibrionales	NA	NA	NA	NA	NA	NA	NA	NA	NA
un_Microbacteriaceae	NA	NA	NA	NA	NA	NA	NA	NA	NA
un_Microbacterium	B	2.17026389	0.04601979	NA	NA	NA	A	2.26489482	0.02472011
un_Micrococcus	NA	NA	NA	NA	NA	NA	NA	NA	NA
un_Microvirga	NA	NA	NA	NA	NA	NA	D	2.33570669	0.00616415
un_Mitochondria	NA	NA	NA	NA	NA	NA	NA	NA	NA
un_Mogibacterium	NA	NA	NA	NA	NA	NA	NA	NA	NA

Supplementary Table 3. LEfSe results for each bacterial taxa throughout vaccination regimen

un_Monoglobus	NA	NA	NA	NA	NA	NA	A	2.99100434	0.04836398
un_Moraxella	NA	NA	NA	NA	NA	NA	D	2.27451798	0.02102774
un_Morganella	NA	NA	NA	NA	NA	NA	NA	NA	NA
un_Mucispirillum	NA	NA	NA	NA	NA	NA	NA	NA	NA
un_Muribaculaceae	NA	NA	NA	A	3.31778925	0.00460402	A	3.34436644	0.0001811
un_Mycobacterium	NA	NA	NA	NA	NA	NA	NA	NA	NA
un_Nautella	NA	NA	NA	C	2.68745015	0.00287642	NA	NA	NA
un_Neisseria	NA	NA	NA	NA	NA	NA	NA	NA	NA
un_Neisseriaceae	NA	NA	NA	NA	NA	NA	D	2.63535204	0.03292446
un_Neptunomonas	NA	NA	NA	C	2.13477639	0.02060563	NA	NA	NA
un_Nesterenkonia	NA	NA	NA	NA	NA	NA	NA	NA	NA
un_Nitrospira	NA	NA	NA	NA	NA	NA	NA	NA	NA
un_Niveispirillum	NA	NA	NA	NA	NA	NA	NA	NA	NA
un_NK4A214_group	NA	NA	NA	NA	NA	NA	A	2.18374439	0.02213389

Supplementary Table 3. LEfSe results for each bacterial taxa throughout vaccination regimen

un_Novosphingobium	NA	NA	NA	NA	NA	NA	D	2.72172376	0.00144211
un_Ochrobactrum	NA	NA	NA	C	3.00783966	0.04405612	NA	NA	NA
un_OM182_clade	NA	NA	NA	NA	NA	NA	NA	NA	NA
un_OM60(NOR5)_clade	NA	NA	NA	NA	NA	NA	NA	NA	NA
un_Oribacterium	NA	NA	NA	NA	NA	NA	NA	NA	NA
un_Oscillibacter	NA	NA	NA	NA	NA	NA	A	1.53975558	0.00616415
un_Oscillospiraceae	NA	NA	NA	A	2.51569579	0.00060936	A	2.5016507	0.00122104
un_Oscillospirales	NA	NA	NA	A	1.59213246	0.00675453	A	1.54099537	0.00616415
un_Oxalobacteraceae	NA	NA	NA	NA	NA	NA	NA	NA	NA
un_Paenibacillus	NA	NA	NA	NA	NA	NA	NA	NA	NA
un_Pantoea	NA	NA	NA	NA	NA	NA	NA	NA	NA
un_Parabacteroides	A	2.02772767	0.0036976	A	2.05679731	0.00055476	NA	NA	NA
un_Paracoccus	NA	NA	NA	NA	NA	NA	NA	NA	NA
un_Parapusillimonas	NA	NA	NA	NA	NA	NA	NA	NA	NA

Supplementary Table 3. LEfSe results for each bacterial taxa throughout vaccination regimen

un_Parasutterella	NA	NA	NA	A	1.531405 14	6.49E-05	A	1.553811 79	0.000243 78
un_Parvimonas	NA	NA	NA	NA	NA	NA	NA	NA	NA
un_Pasteurellaceae	NA	NA	NA	NA	NA	NA	D	1.990015 92	0.011701 28
un_Pediococcus	NA	NA	NA	NA	NA	NA	D	2.317810 28	0.003227 6
un_Pedobacter	NA	NA	NA	NA	NA	NA	NA	NA	NA
un_Pelomonas	NA	NA	NA	NA	NA	NA	NA	NA	NA
un_Peptoniphilus	NA	NA	NA	C	2.694921 37	0.037835 52	NA	NA	NA
un_Peptostreptococcaceae	NA	NA	NA	A	1.529028 77	0.023665 49	NA	NA	NA
un_Peptostreptococcus	NA	NA	NA	NA	NA	NA	NA	NA	NA
un_Perlucidibaca	NA	NA	NA	NA	NA	NA	NA	NA	NA
un_Phascolarctobacterium	NA	NA	NA	NA	NA	NA	NA	NA	NA
un_Phenylobacterium	NA	NA	NA	NA	NA	NA	NA	NA	NA
un_Phreatobacter	NA	NA	NA	NA	NA	NA	NA	NA	NA
un_Planktomarina	NA	NA	NA	NA	NA	NA	NA	NA	NA
un_Planococcaceae	NA	NA	NA	NA	NA	NA	D	2.203738 44	0.003227 6

Supplementary Table 3. LEfSe results for each bacterial taxa throughout vaccination regimen

un_Porphyromonas	NA	NA	NA	NA	NA	NA	NA	NA	NA
un_Prevotella	NA	NA	NA	NA	NA	NA	NA	NA	NA
un_Prevotellaceae	NA	NA	NA	A	1.56282161	0.00358085	A	1.64798216	0.0032276
un_Prevotellaceae_UCG-001	NA	NA	NA	A	1.5459216	0.00025846	A	1.61221274	0.00022146
un_probable_genus_10	NA	NA	NA	NA	NA	NA	NA	NA	NA
un_Proteobacteria	NA	NA	NA	NA	NA	NA	NA	NA	NA
un_Providencia	B	2.8045647	0.02954715	NA	NA	NA	NA	NA	NA
un_PS1_clade	NA	NA	NA	NA	NA	NA	NA	NA	NA
un_Pseudarcobacter	NA	NA	NA	C	1.8115938	0.01074057	D	1.81359032	0.01170128
un_Pseudarthrobacter	B	2.4619213	0.0277698	NA	NA	NA	NA	NA	NA
un_Pseudobutyrivibrio	NA	NA	NA	NA	NA	NA	NA	NA	NA
un_Pseudoclavibacter	NA	NA	NA	NA	NA	NA	NA	NA	NA
un_Pseudomonas	NA	NA	NA	NA	NA	NA	NA	NA	NA
un_Pseudonocardia	NA	NA	NA	NA	NA	NA	NA	NA	NA
un_Pseudopropionibacterium	NA	NA	NA	NA	NA	NA	NA	NA	NA
un_Pseudoxanthomonas	NA	NA	NA	NA	NA	NA	NA	NA	NA

Supplementary Table 3. LEfSe results for each bacterial taxa throughout vaccination regimen

un_Psychrobacter	NA	NA	NA	NA	NA	NA	NA	NA	NA
un_Quinella	NA	NA	NA	A	1.98734425	1.54E-05	A	2.04385277	1.26E-05
un_Ralstonia	A	1.92202193	0.01176743	A	1.93449878	0.00716867	A	1.95435694	0.00621798
un_Rheinheimera	NA	NA	NA	NA	NA	NA	NA	NA	NA
un_Rhizobiaceae	NA	NA	NA	NA	NA	NA	D	2.46552165	0.00018966
un_Rhodobacter	NA	NA	NA	NA	NA	NA	NA	NA	NA
un_Rhodobacteraceae	B	1.56214304	0.00223374	C	2.03248664	0.00079408	D	2.09600233	0.0083756
un_Rhodococcus	NA	NA	NA	NA	NA	NA	NA	NA	NA
un_Rikenellaceae	NA	NA	NA	NA	NA	NA	NA	NA	NA
un_Rikenellaceae_RC9_gut_group	NA	NA	NA	NA	NA	NA	NA	NA	NA
un_Romboutsia	NA	NA	NA	A	3.14108133	0.00015507	A	3.1928263	0.00010539
un_Roseburia	NA	NA	NA	NA	NA	NA	A	2.1505901	0.00538758
un_Roseobacter_clade_CHAB-I-5_lineage	NA	NA	NA	NA	NA	NA	NA	NA	NA
un_Roseomonas	NA	NA	NA	NA	NA	NA	NA	NA	NA
un_Rothia	NA	NA	NA	NA	NA	NA	NA	NA	NA

Supplementary Table 3. LEfSe results for each bacterial taxa throughout vaccination regimen

un_Rubellimicrobium	NA	NA	NA	NA	NA	NA	NA	NA	NA
un_Ruminococcaceae	NA	NA	NA	NA	NA	NA	NA	NA	NA
un_Ruminococcus	NA	NA	NA	NA	NA	NA	NA	NA	NA
un_Saccharimonadaceae	NA	NA	NA	NA	NA	NA	NA	NA	NA
un_Saccharimonadaceae	NA	NA	NA	NA	NA	NA	NA	NA	NA
un_Saccharofermentans	NA	NA	NA	NA	NA	NA	A	2.33805815	0.02213389
un_Sandaracinobacter	NA	NA	NA	C	1.87677057	0.02060563	NA	NA	NA
un_SC-I-84	NA	NA	NA	NA	NA	NA	NA	NA	NA
un_Schlegelella	NA	NA	NA	NA	NA	NA	NA	NA	NA
un_SD04E11	NA	NA	NA	NA	NA	NA	NA	NA	NA
un_Selenomonas	NA	NA	NA	NA	NA	NA	NA	NA	NA
un_Sericytochromatia	NA	NA	NA	NA	NA	NA	NA	NA	NA
un_Serratia	NA	NA	NA	C	2.96193836	0.03437702	D	3.84360032	0.00154353
un_Shewanella	B	2.03723364	0.0127706	NA	NA	NA	NA	NA	NA
un_Shinella	NA	NA	NA	NA	NA	NA	NA	NA	NA
un_Silvanigrellaceae	NA	NA	NA	NA	NA	NA	NA	NA	NA

Supplementary Table 3. LEfSe results for each bacterial taxa throughout vaccination regimen

un_Skermanella	NA	NA	NA	NA	NA	NA	D	1.7432485	0.00010938
un_Solirubrobacter	NA	NA	NA	NA	NA	NA	NA	NA	NA
un_Solobacterium	NA	NA	NA	NA	NA	NA	NA	NA	NA
un_Sphingobacterium	B	3.32392756	0.00421732	NA	NA	NA	NA	NA	NA
un_Sphingobium	NA	NA	NA	NA	NA	NA	NA	NA	NA
un_Sphingomonadaceae	B	1.70271554	0.0304467	NA	NA	NA	NA	NA	NA
un_Sphingomonas	B	3.3632511	0.00302165	NA	NA	NA	D	3.71939699	0.00755136
un_Staphylococcus	NA	NA	NA	NA	NA	NA	NA	NA	NA
un_Stenotrophomonas	NA	NA	NA	NA	NA	NA	NA	NA	NA
un_Stomatobaculum	NA	NA	NA	NA	NA	NA	NA	NA	NA
un_Streptobacillus	NA	NA	NA	NA	NA	NA	NA	NA	NA
un_Streptococcus	NA	NA	NA	NA	NA	NA	NA	NA	NA
un_Subdoligranulum	NA	NA	NA	NA	NA	NA	NA	NA	NA
un_Succiniclasticum	NA	NA	NA	NA	NA	NA	NA	NA	NA
un_Succinivibrionaceae	NA	NA	NA	NA	NA	NA	NA	NA	NA
un_SUP05_cluster	NA	NA	NA	A	2.51071936	0.0442347	A	2.61784107	0.04187977

Supplementary Table 3. LEfSe results for each bacterial taxa throughout vaccination regimen

un_Sutterella	NA	NA	NA	A	2.13324034	0.0442347	NA	NA	NA
un_Tepidimonas	NA	NA	NA	NA	NA	NA	D	3.27150732	0.01962395
un_Tepidiphilus	NA	NA	NA	NA	NA	NA	NA	NA	NA
un_Terrisporobacter	NA	NA	NA	NA	NA	NA	NA	NA	NA
un_Thermicanus	NA	NA	NA	NA	NA	NA	NA	NA	NA
un_Thioglobaceae	NA	NA	NA	NA	NA	NA	NA	NA	NA
un_TM7a	NA	NA	NA	NA	NA	NA	NA	NA	NA
un_TM7x	NA	NA	NA	NA	NA	NA	NA	NA	NA
un_Treponema	NA	NA	NA	A	1.90654783	0.01598344	A	1.84907635	0.0032276
un_Tsukamurella	NA	NA	NA	NA	NA	NA	NA	NA	NA
un_Turicibacter	A	3.35424748	0.03089949	A	3.26080153	1.61E-06	A	3.33130368	1.25E-06
un_Tuzzerella	NA	NA	NA	NA	NA	NA	NA	NA	NA
un_UBA1819	NA	NA	NA	NA	NA	NA	NA	NA	NA
un_UCG-002	NA	NA	NA	NA	NA	NA	NA	NA	NA
un_UCG-003	NA	NA	NA	NA	NA	NA	NA	NA	NA
un_UCG-005	NA	NA	NA	NA	NA	NA	NA	NA	NA
un_UCG-010	NA	NA	NA	NA	NA	NA	NA	NA	NA

Supplementary Table 3. LEfSe results for each bacterial taxa throughout vaccination regimen

un_Uruburuella	NA	NA	NA	NA	NA	NA	NA	NA	NA
un_Veillonella	NA	NA	NA	NA	NA	NA	NA	NA	NA
un_Vibrio	NA	NA	NA	NA	NA	NA	NA	NA	NA
un_Vicinamibacteraceae	NA	NA	NA	NA	NA	NA	NA	NA	NA
un_Vogesella	NA	NA	NA	C	2.02898257	0.02060563	NA	NA	NA
un_Vulcaniibacterium	NA	NA	NA	NA	NA	NA	NA	NA	NA
un_Weissella	NA	NA	NA	NA	NA	NA	NA	NA	NA
un_Williamsia	NA	NA	NA	NA	NA	NA	NA	NA	NA
un_Xanthobacter	NA	NA	NA	NA	NA	NA	NA	NA	NA
un_Xanthobacteraceae	NA	NA	NA	NA	NA	NA	NA	NA	NA
unidentified_Deinococcus	NA	NA	NA	NA	NA	NA	NA	NA	NA
unidentified_Muribaculaceae	A	1.55156251	0.03662603	A	1.51327094	0.00358085	A	1.57432567	0.0032276
unidentified_Odoribacter	NA	NA	NA	A	1.51624338	0.0001303	A	1.57531973	0.00011022
unidentified_rumen_Prevotella	NA	NA	NA	NA	NA	NA	NA	NA	NA
unidentified_rumen_Treponema	NA	NA	NA	NA	NA	NA	NA	NA	NA
Veillonella_atypica	NA	NA	NA	NA	NA	NA	NA	NA	NA
Veillonella_sp.	NA	NA	NA	NA	NA	NA	NA	NA	NA
Vibrio_diazotrophicus	NA	NA	NA	NA	NA	NA	NA	NA	NA

Supplementary Table 3. LEfSe results for each bacterial taxa throughout vaccination regimen

Vibrio_fluvialis	NA	NA	NA	NA	NA	NA	NA	NA	NA
Vibrio_navarrensis	NA	NA	NA	NA	NA	NA	NA	NA	NA
Winkia_neuii	NA	NA	NA	NA	NA	NA	NA	NA	NA
Xanthomonas_sp.	NA	NA	NA	NA	NA	NA	NA	NA	NA

Note. Sample size, Baseline: 44, One week after 1st dose: 44, One week after 2nd dose: 43, One month after second dose: 44

Supplementary Table 4. Mixed effect models for persistently differentially abundant species between subjects with high- and low-IgA levels

Features	Model 1		Model 2		Model 3	
	Effect size	P value	Effect size	P value	Effect size	P value
Actinomyces massiliensis	1e-04 (-3.77e-05, 2.38e-04)	0.15	1.21e-04 (-1.77e-05, 2.60e-04)	0.086	1.24e-04 (-1.48e-05, 2.63e-04)	0.078
Corynebacterium kroppenstedtii	-1.94e-02 (-5.06e-02, 1.18e-02)	0.217	-1.68e-02 (-4.82e-02, 1.46e-02)	0.287	-1.64e-02 (-4.82e-02, 1.53e-02)	0.301
Cutibacterium granulosum	1.88e-03 (-1.51e-04, 3.90e-03)	0.069	1.89e-03 (-1.95e-04, 3.97e-03)	0.074	1.72e-03 (-3.26e-04, 3.77e-03)	0.097
Endobacter medicaginis	2.63e-05 (-2.47e-06, 5.51e-05)	0.072	2.64e-05 (-3.22e-06, 5.59e-05)	0.079	2.67e-05 (-3.12e-06, 5.65e-05)	0.078
Fusobacterium sp.	8.32e-06 (-1.65e-05, 3.31e-05)	0.502	9.07e-06 (-1.63e-05, 3.45e-05)	0.475	7.91e-06 (-1.74e-05, 3.32e-05)	0.531
Neisseria elongata	1.36e-04 (1.31e-05, 2.60e-04)	0.031	1.3e-04 (3.76e-06, 2.56e-04)	0.044	1.31e-04 (3.44e-06, 2.59e-04)	0.044
Prevotella histicola	1.06e-03 (-2.51e-04, 2.38e-03)	0.11	1.22e-03 (-9.23e-05, 2.53e-03)	0.068	1.26e-03 (-5.60e-05, 2.58e-03)	0.06
Pseudomonas balearica	1.43e-03 (-2.10e-04, 3.08e-03)	0.086	1.68e-03 (5.4e-05, 3.3e-03)	0.043	1.67e-03 (9.74e-05, 3.25e-03)	0.038
Ruminococcus callidus	1.56e-05 (-2.01e-05, 5.13e-05)	0.381	1.31e-05 (-2.32e-05, 4.94e-05)	0.47	1.16e-05 (-2.50e-05, 4.83e-05)	0.524
unclassified Acinetobacter	2.59e-03 (-6.44e-03, 1.16e-02)	0.565	2.7e-03 (-6.59e-03, 1.20e-02)	0.56	3.01e-03 (-6.22e-03, 1.22e-02)	0.514
unclassified Actinobacillus	1.06e-04 (-9.21e-05, 3.05e-04)	0.286	1.47e-04 (-4.85e-05, 3.43e-04)	0.136	1.52e-04 (-4.62e-05, 3.50e-04)	0.129
unclassified Aggregatibacter	1.91e-04 (-1.05e-05, 3.93e-04)	0.063	2.19e-04 (1.90e-05, 4.18e-04)	0.033	2.25e-04 (2.84e-05, 4.21e-04)	0.026
unclassified Alishewanella	9.59e-05 (-2.82e-06, 1.95e-04)	0.057	1.14e-04 (1.9e-05, 2.1e-04)	0.02	1.17e-04 (2.49e-05, 2.10e-04)	0.014
unclassified Brevundimonas	-4.68e-04 (-1.02e-03, 8.65e-05)	0.096	-4.24e-04 (-9.89e-04, 1.42e-04)	0.138	-4.34e-04 (-1.01e-03, 1.41e-04)	0.134
unclassified Castellaniella	3.16e-03 (-5.85e-04, 6.91e-03)	0.096	3.04e-03 (-8.04e-04, 6.89e-03)	0.118	3.06e-03 (-8.46e-04, 6.97e-03)	0.121
unclassified Clostridium sensu stricto 1	2.56e-03 (-1.70e-03, 6.82e-03)	0.232	2.94e-03 (-1.41e-03, 7.28e-03)	0.179	2.69e-03 (-1.61e-03, 6.98e-03)	0.213

unclassified Incertae Sedis						
Incertae Sedis	6.45e-06 (1.88e-08, 1.29e-05)	0.049	6.29e-06 (-3.10e-07, 1.29e-05)	0.061	6.27e-06 (-3.72e-07, 1.29e-05)	0.064
Ruminococcaceae						
unclassified Monoglobus	1.54e-03 (-9.76e-04, 4.06e-03)	0.223	1.52e-03 (-9.83e-04, 4.03e-03)	0.227	1.61e-03 (-9.21e-04, 4.13e-03)	0.206
unclassified Neisseria	1.29e-02 (2.78e-04, 2.55e-02)	0.045	1.28e-02 (2.80e-04, 2.54e-02)	0.045	1.27e-02 (-3.70e-04, 2.58e-02)	0.056
unclassified Pseudopropionibacterium	2.61e-04 (9.71e-07, 5.22e-04)	0.049	2.61e-04 (2.46e-06, 5.20e-04)	0.048	3.02e-04 (4.38e-05, 5.60e-04)	0.023
unclassified Skermanella	4.07e-05 (-1.69e-05, 9.83e-05)	0.161	4.23e-05 (-1.66e-05, 1.01e-04)	0.155	4.66e-05 (-1.18e-05, 1.05e-04)	0.114
unclassified Stomatobaculum	1.37e-05 (-9.99e-06, 3.73e-05)	0.25	1.36e-05 (-9.92e-06, 3.71e-05)	0.25	1.5e-05 (-9.24e-06, 3.93e-05)	0.217
unclassified Sutterella	2.54e-04 (-1.47e-04, 6.55e-04)	0.208	2.51e-04 (-1.48e-04, 6.50e-04)	0.212	2.68e-04 (-1.35e-04, 6.70e-04)	0.186
unclassified Terrisporobacter	3.87e-05 (-2.17e-05, 9.92e-05)	0.203	3.82e-05 (-2.20e-05, 9.84e-05)	0.207	3.66e-05 (-2.49e-05, 9.81e-05)	0.236

Model 1 is a crude model; Model 2 is adjusted for age and time difference between two doses. Model 3 is adjusted for age and time difference between two doses, epidural anesthesia, and intramuscular analgesia. Sample size: 43; High IgA: 18, Low IgA: 25

Supplementary Table 5. LEfSe results for each probiotic taxa (*Lactobacilli & Bifidobacteria*) throughout vaccination regimen

Features	group1	group2	n1	n2	statistic	p	Adju	p.adj.signif	A (min, max)	B (min, max)	C (min, max)	D (min, max)	A [Median (IQR)]	B [Median (IQR)]	C [Median (IQR)]	D [Median (IQR)]
Lactobacillus aviarius	A	B	44	44	2.486831	0.012889	0.038666	*	(0, 0.064)	(0, 0.015)	(0, 6.00E-04)	(0, 7.18E-04)	0 (0, 6.72E-04)	2.38E-04 (0, 5.51E-04)	0 (0, 0)	0 (0, 0)
	A	C	44	43	-2.43783	0.014776	0.038666	*								
	A	D	44	44	-3.14511	0.00166	0.006641	**								
	B	C	44	43	-4.91033	9.09E-07	4.55E-06	****								
	B	D	44	44	-5.63194	1.78E-08	1.07E-07	****								
	C	D	43	44	-0.68915	0.490727	0.490727	ns								
Lactobacillus fermentum	A	B	44	44	0.744194	0.456759	0.913518	ns	(0, 6.79E-05)	(0, 2.27E-04)	(0, 8.71E-05)	(0, 7.60E-04)	0 (0, 0)	0 (0, 0)	0 (0, 0)	0 (0, 0)
	A	C	44	43	1.363549	0.17271	0.518129	ns								
	A	D	44	44	3.037168	0.002388	0.014329	*								
	B	C	44	43	0.623644	0.532861	0.913518	ns								
	B	D	44	44	2.292975	0.021849	0.109247	ns								
	C	D	43	44	1.656114	0.097699	0.390795	ns								
Lactobacillus gasseri	A	B	44	44	1.225184	0.220506	1	ns	(0, 0.028)	(0, 0.030)	(0, 0.381)	(0, 0.034)	0 (0, 3.10E-04)	4.33E-05 (0, 4.44E-04)	1.73E-05 (0, 3.51E-04)	1.75E-05 (0, 6.70E-04)
	A	C	44	43	0.981653	0.326271	1	ns								
	A	D	44	44	1.186897	0.235268	1	ns								
	B	C	44	43	-0.23647	0.813068	1	ns								
	B	D	44	44	-0.03829	0.969459	1	ns								
	C	D	43	44	0.198403	0.84273	1	ns								

Lactobacillus iners	A	B	44	44	2.193275	0.028288	0.11315	ns	(0, 8.58E-05)	(0, 0.001)	(0, 2.11E-04)	(0, 5.60E-05)	0 (0, 0)	0 (0, 0)	0 (0, 0)	0 (0, 0)
	A	C	44	43	-0.3998	0.689303	1	ns								
	A	D	44	44	-0.42765	0.668905	1	ns								
	B	C	44	43	-2.58044	0.009868	0.052615	ns								
	B	D	44	44	-2.62093	0.008769	0.052615	ns								
	C	D	43	44	-0.02539	0.979748	1	ns								
Lactobacillus intestinalis	A	B	44	44	-2.09884	0.035831	0.214988	ns	(0, 0.001)	(0, 0)	(0, 3.83E-04)	(0, 1.86E-03)	0 (0, 0)	0 (0, 0)	0 (0, 0)	0 (0, 0)
	A	C	44	43	-0.81144	0.417112	1	ns								
	A	D	44	44	-0.40824	0.683097	1	ns								
	B	C	44	43	1.275298	0.202204	0.808815	ns								
	B	D	44	44	1.690596	0.090914	0.45457	ns								
	C	D	43	44	0.405554	0.68507	1	ns								
Lactobacillus sp.	A	B	44	44	-0.04574	0.963517	0.963517	ns	(0, 0.048)	(0, 0.015)	(0, 9.24E-04)	(0, 0.006)	1.35E-04 (0, 5.30E-04)	1.20E-04 (0, 6.07E-04)	1.72E-05 (0, 1.01E-04)	4.37E-05 (0, 3.01E-04)
	A	C	44	43	-2.39793	0.016488	0.098929	ns								
	A	D	44	44	-1.35696	0.174793	0.699172	ns								
	B	C	44	43	-2.35245	0.01865	0.098929	ns								
	B	D	44	44	-1.31122	0.189783	0.699172	ns								
	C	D	43	44	1.048784	0.294278	0.699172	ns								
Bifidobacterium bifidum	A	B	44	44	-1.55649	0.119592	0.597959	ns	(0, 2.49E-03)	(0, 1.77E-04)	(0, 5.58E-04)	(0, 1.09E-03)	0 (0,0)	0 (0,0)	0 (0,0)	0 (0,0)
	A	C	44	43	-1.50243	0.132986	0.597959	ns								
	A	D	44	44	-2.30343	0.021255	0.12753	ns								
	B	C	44	43	0.045089	0.964036	1	ns								

	B	D	44	44	-0.74694	0.455102	1	ns								
	C	D	43	44	-0.78772	0.43086	1	ns								
Bifidobacterium breve	A	B	44	44	-0.07532	0.939963	1	ns	(0,0.034)	(0,0.005)	(0,0.005)	(0,0.050)	0 (0, 2.12E-04)	0 (0, 1.53E-04)	0 (0, 7.45E-05)	0 (0,9.00E-05)
	A	C	44	43	-1.08361	0.278538	1	ns								
	A	D	44	44	-0.63845	0.52318	1	ns								
	B	C	44	43	-1.00873	0.313106	1	ns								
	B	D	44	44	-0.56313	0.573343	1	ns								
	C	D	43	44	0.448838	0.653549	1	ns								
Bifidobacterium dentium	A	B	44	44	-2.06894	0.038552	0.231312	ns	(0,0.028)	(0, 8.52E-04)	(0,0.002)	(0,0.007)	0 (0,0)	0 (0,0)	0 (0,0)	0 (0,0)
	A	C	44	43	-2.05697	0.039689	0.231312	ns								
	A	D	44	44	-1.75918	0.078547	0.314187	ns								
	B	C	44	43	4.5E-05	0.999964	1	ns								
	B	D	44	44	0.309756	0.756746	1	ns								
	C	D	43	44	0.307926	0.758139	1	ns								
Bifidobacterium longum	A	B	44	44	-0.57647	0.564296	1	ns	(0,0.004)	(0, 5.53E-04)	(0, 0.006)	(0, 2.91E-03)	0 (0, 7.01E-05)	0 (0, 2.71E-05)	0 (0,0)	0 (0,0)
	A	C	44	43	-1.41932	0.155806	0.934834	ns								
	A	D	44	44	-1.21839	0.223076	1	ns								
	B	C	44	43	-0.84617	0.397458	1	ns								
	B	D	44	44	-0.64192	0.520927	1	ns								
	C	D	43	44	0.207953	0.835266	1	ns								
Bifidobacterium sp.	A	B	44	44	0.874366	0.381919	1	ns	(0,0.009)	(0,0.009)	(0,0.022)	(0,0.007)		2.27E-04 (5.62E-	1.38E-04 (7.43E-	
	A	C	44	43	0.071004	0.943394	1	ns								

	A	D	44	44	-0.90196	0.367081	1	ns					1.79E-04 (0, 6.92E-04)	05,8.69E-04)	05,3.99E-04)	1.15E-04 (0, 4.31E-04)
	B	C	44	43	-0.79832	0.424684	1	ns								
	B	D	44	44	-1.77632	0.07568	0.45408	ns								
	C	D	43	44	-0.96776	0.333164	1	ns								

Sample size, Baseline (A): 44, One week after 1st dose (B): 44, One week after 2nd dose (C): 43, One month after second dose (D): 44

Supplementary Table 6. Mixed effect models for persistently differentially abundant species in *Lactobacillus* and *Bifidobacterium* between subjects with high- and low-IgA levels

	Model 1		Model 2		Model 3	
Features	Effect size	P value	Effect size	P value	Effect size	P value
Bifidobacterium bifidum	1.88e-04 (-5.30e-05, 4.29e-04)	0.123	1.72e-04 (-7.72e-05, 4.21e-04)	0.171	1.38e-04 (-1.39e-04, 4.15e-04)	0.318
Bifidobacterium breve	-1.48e-03 (-4.76e-03, 1.80e-03)	0.368	-1.58e-03 (-4.99e-03, 1.83e-03)	0.354	-9.34e-04 (-4.70e-03, 2.84e-03)	0.618
Bifidobacterium dentium	-9.94e-04 (-3.64e-03, 1.65e-03)	0.452	-9.54e-04 (-3.69e-03, 1.79e-03)	0.485	-7.42e-04 (-3.76e-03, 2.28e-03)	0.621
Bifidobacterium longum	2.23e-04 (-1.73e-04, 6.18e-04)	0.262	2.15e-04 (-1.96e-04, 6.26e-04)	0.296	1.82e-04 (-2.72e-04, 6.36e-04)	0.421
Lactobacillus aviarius	3.05e-03 (-3.08e-03, 9.18e-03)	0.321	2.85e-03 (-3.52e-03, 9.22e-03)	0.371	2.12e-03 (-4.89e-03, 9.12e-03)	0.543
Lactobacillus fermentum	-2.72e-06 (-9.20e-06, 3.77e-06)	0.403	-3.06e-06 (-9.75e-06, 3.62e-06)	0.36	-3.9e-06 (-1.14e-05, 3.61e-06)	0.299
Lactobacillus gasseri	-1.39e-03 (-4.12e-03, 1.34e-03)	0.311	-1.09e-03 (-3.86e-03, 1.69e-03)	0.433	-9.12e-04 (-3.91e-03, 2.08e-03)	0.54
Lactobacillus iners	-4.14e-06 (-1.24e-05, 4.16e-06)	0.32	-3.83e-06 (-1.24e-05, 4.76e-06)	0.373	-5.38e-06 (-1.49e-05, 4.16e-06)	0.26
Lactobacillus intestinalis	1.11e-04 (-2.15e-05, 2.44e-04)	0.098	1.23e-04 (-1.22e-05, 2.58e-04)	0.073	9.04e-05 (-4.89e-05, 2.30e-04)	0.196
Unclassified Bifidobacterium	3.75e-04 (-8.13e-04, 1.56e-03)	0.528	3.98e-04 (-8.38e-04, 1.63e-03)	0.519	2.08e-04 (-1.18e-03, 1.59e-03)	0.763
Unclassified Lactobacillus	3.11e-03 (-1.75e-03, 7.97e-03)	0.203	3.48e-03 (-1.53e-03, 8.49e-03)	0.167	2.12e-03 (-3.18e-03, 7.41e-03)	0.423

Model 1 is a crude model; Model 2 is adjusted for age and time difference between two doses; Model 3 is adjusted for age and time difference between two doses, epidural anesthesia, and intramuscular analgesia. Sample size: 43; High IgA: 18, Low IgA: 25

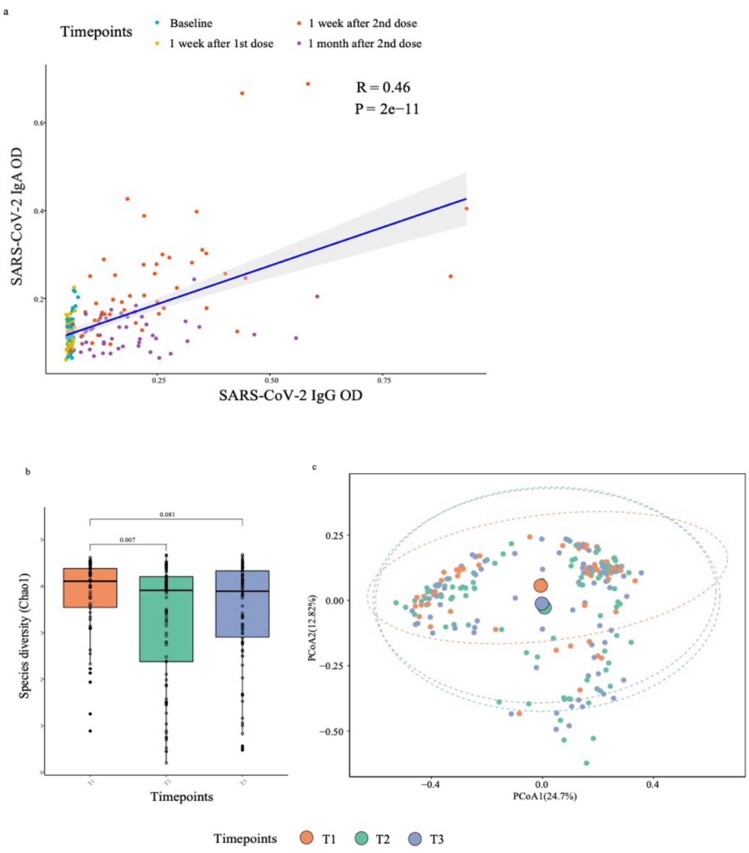

PERMANOVA	F value	Adjusted P value
T1 vs T2	1.96	0.240
T1 vs T3	1.41	0.360
T2 vs T3	0.25	1.000

Supplementary Figure 1. Correlation plot for immunity parameters and alpha diversity for the control cohort. (A) The correlation plot between immunoglobulin A and immunoglobulin G in breast milk over different timepoints (Sample size, Baseline: 49, One week after 1st dose: 48, One week after 2nd dose: 48, One month after second dose: 48). The Spearman's correlation coefficient (rho value) and corresponding *p* value are shown. (B) The species diversity and Chao1 index was compared using Wilcoxon signed-rank test over the first three months postpartum at three time points: Week 4 (T1), Week 8 (T2), and Week 12 (T3) (Sample size, 110). Elements on boxplots: centre line, median; box limits, upper and lower quartiles; whiskers, 1.5×IQR.

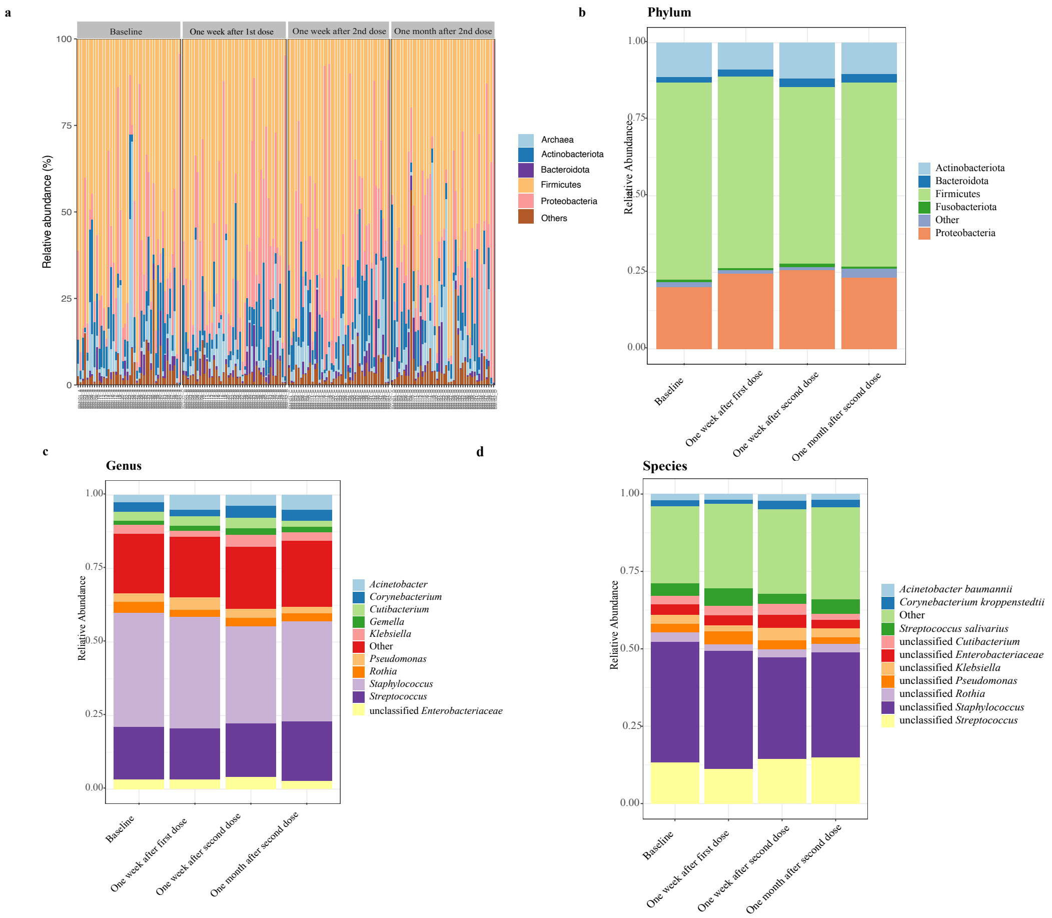

Supplementary Figure 2. Compositional differences in breast milk microbiota of lactating mothers before and after vaccination at baseline, one week post-first dose, one week post-second dose, and one month post-second dose of BNT162b2 (Sample size, Baseline: 44, One week after 1st dose: 44, One week after 2nd dose: 43, One month after second dose: 44). (A) Breast milk microbiota profile at the phylum level across samples. (B) Breast milk microbiota profile at the phylum level across timepoints. (C) Breast milk microbiota profile at the genus level across timepoints. (D) Breast milk microbiota profile at the species level across timepoints.

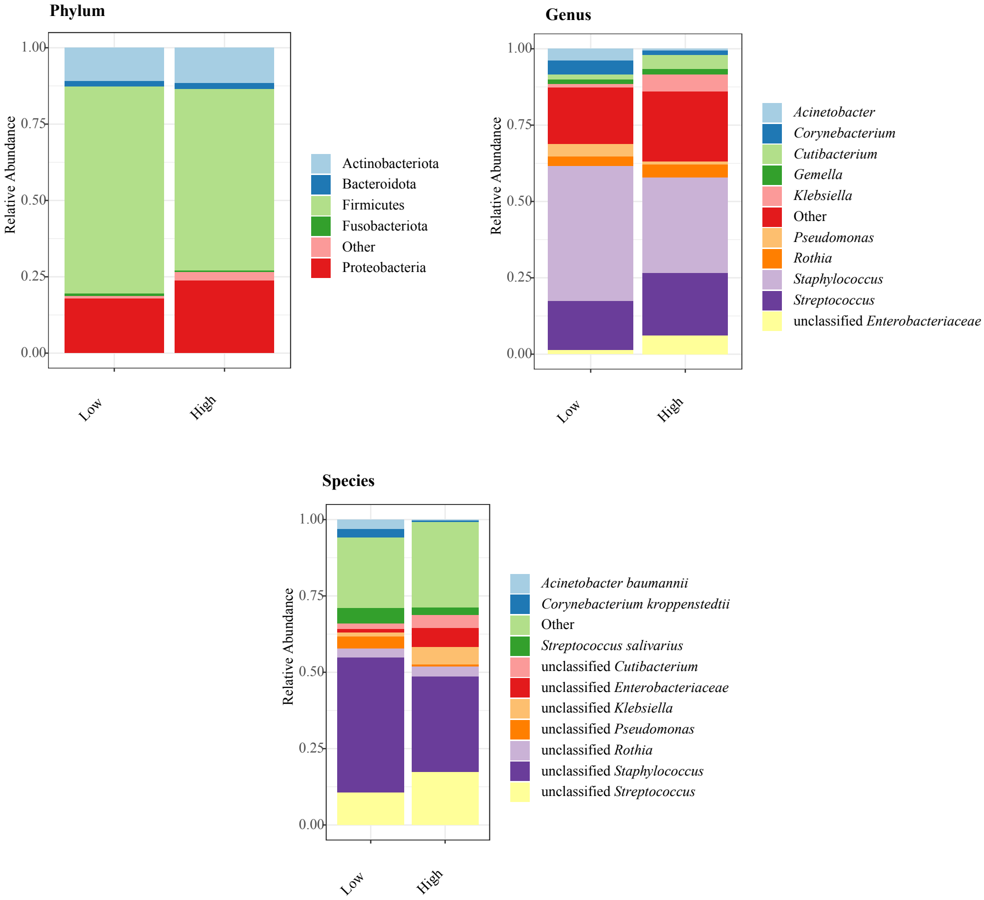

Supplementary Figure 3. Compositional differences in baseline breast milk microbiota of lactating mothers with high- and low-IgA subjects to BNT162b2 vaccination one week post-second dose (N=43; High IgA: 18, Low IgA: 25). (A) Breast milk microbiota profile at the phylum level across timepoints. (C) Breast milk microbiota profile at the genus level across timepoints. (D) Breast milk microbiota profile at the species level across timepoints.

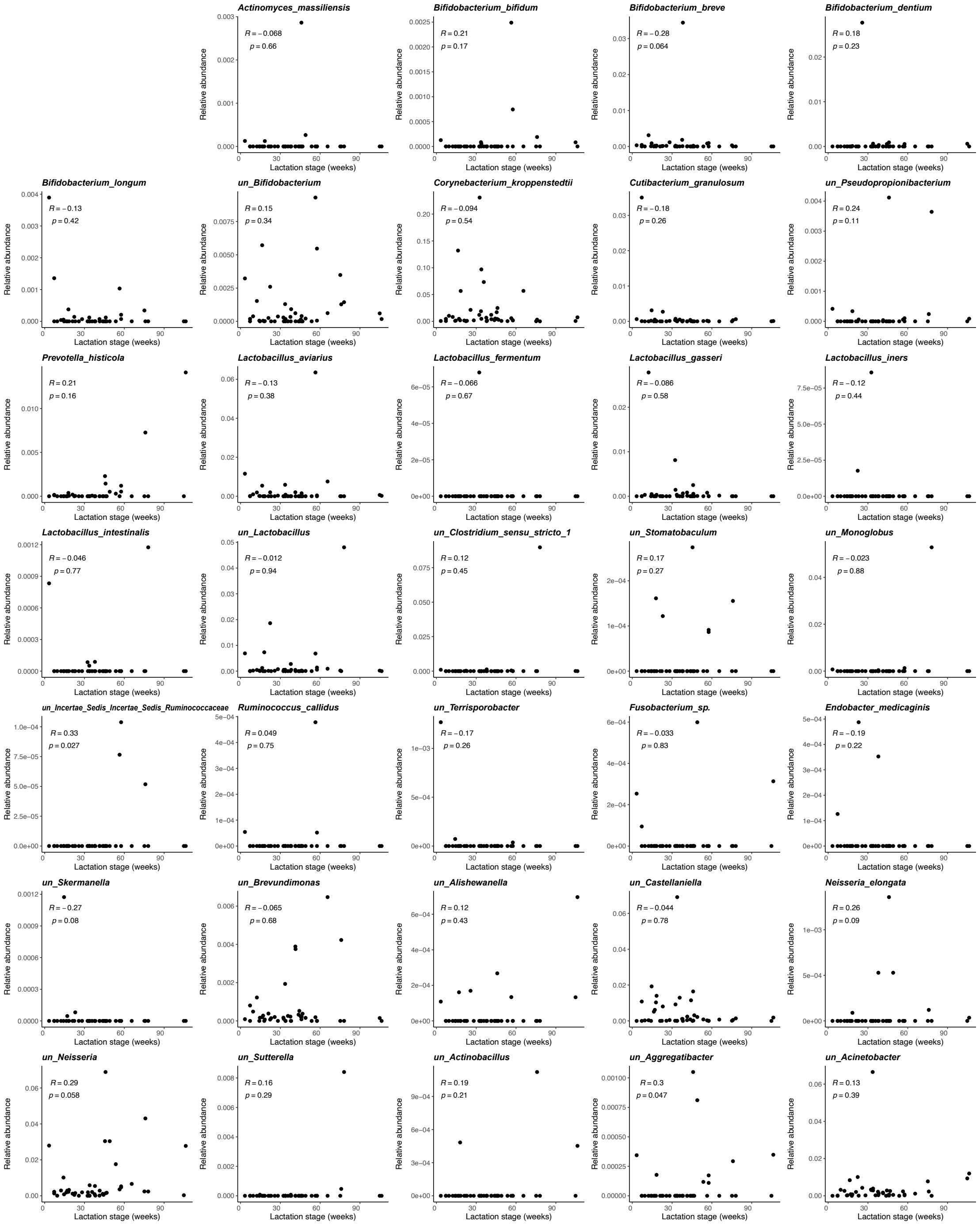

Supplementary Figure 4. The correlation plot between lactation stage at baseline (weeks) and relative abundance of baseline markers from LEfSe and probiotics in breast milk (Sample size, Baseline: 44). The Spearman's correlation coefficient (rho value) and corresponding *p* value are shown.
