# Supplementary material for: COVID-19 mRNA vaccine-mediated antibodies in human breast milk and their association with breast milk microbiota composition
Source: NPJ Vaccines. 2023 Oct 5;8:151. doi: 10.1038/s41541-023-00745-4 (PMC10556030; doi:10.1038/s41541-023-00745-4)
Supplement: Supplementary file 2 — Reporting Summary [file 41541_2023_745_MOESM2_ESM.pdf]

nature portfolio

Corresponding author(s): Hein Tun

Last updated by author(s): Sep 8, 2023

Reporting Summary

Nature Portfolio wishes to improve the reproducibility of the work that we publish. This form provides structure for consistency and transparency in reporting. For further information on Nature Portfolio policies, see our Editorial Policies and the Editorial Policy Checklist.

Statistics

For all statistical analyses, confirm that the following items are present in the figure legend, table legend, main text, or Methods section.

n/a	Confirmed	
☐	☒	The exact sample size (*n*) for each experimental group/condition, given as a discrete number and unit of measurement
☐	☒	A statement on whether measurements were taken from distinct samples or whether the same sample was measured repeatedly
☐	☒	The statistical test(s) used AND whether they are one- or two-sided<br>*Only common tests should be described solely by name; describe more complex techniques in the Methods section.*
☐	☒	A description of all covariates tested
☐	☒	A description of any assumptions or corrections, such as tests of normality and adjustment for multiple comparisons
☐	☒	A full description of the statistical parameters including central tendency (e.g. means) or other basic estimates (e.g. regression coefficient) AND variation (e.g. standard deviation) or associated estimates of uncertainty (e.g. confidence intervals)
☐	☒	For null hypothesis testing, the test statistic (e.g. *F*, *t*, *r*) with confidence intervals, effect sizes, degrees of freedom and *P* value noted<br>*Give P values as exact values whenever suitable.*
☒	☐	For Bayesian analysis, information on the choice of priors and Markov chain Monte Carlo settings
☒	☐	For hierarchical and complex designs, identification of the appropriate level for tests and full reporting of outcomes
☐	☒	Estimates of effect sizes (e.g. Cohen's *d*, Pearson's *r*), indicating how they were calculated

Our web collection on statistics for biologists contains articles on many of the points above.

Software and code

Policy information about availability of computer code

Data collection	In total of 49 lactating mothers who planned to receive the BNT162b2 mRNA vaccine, when more than 99% of the Hong Kong population was not infected by COVID-19. Breast milk was self-collected at the participants' home in sterile milk bags at four timepoints (pre-vaccination, one week post-first dose, one week post-second dose, and one month post-second dose). SARS-CoV-2 spike protein-specific IgA and IgG levels in breast milk were measured to assess immune responses to BNT162b2 at different stages of the vaccination regimen using ELISA. DNA was extracted from breast milk using QIAamp PowerFecal Pro DNA Kit (QIAGEN, Hilden, Germany). V3-V4 regions of the 16S rRNA genes were amplified and sequenced by Illumina MiSeq.
Data analysis	Unless otherwise specified, all analyses and data visualization were performed in R V4.2.0. Two-sided values of p values < 0.05 were considered statistically significant. Multivariate analysis by the linear models (MaAsLin2) statistical framework was implemented to evaluate the association between specific microbial markers and demographical characteristics in the Huttenhower Lab Galaxy instance (http://huttenhower.sph.harvard.edu/galaxy/). The p values for metagenomics analyses were adjusted for multiple comparisons using FDR correction.

For manuscripts utilizing custom algorithms or software that are central to the research but not yet described in published literature, software must be made available to editors and reviewers. We strongly encourage code deposition in a community repository (e.g. GitHub). See the Nature Portfolio guidelines for submitting code & software for further information.

Data

Policy information about availability of data

All manuscripts must include a data availability statement. This statement should provide the following information, where applicable:

- Accession codes, unique identifiers, or web links for publicly available datasets
- A description of any restrictions on data availability
- For clinical datasets or third party data, please ensure that the statement adheres to our policy

Sequencing reads were deposited at Sequence Read Archive under accession PRJNA917338. Additional datasets generated and/or analysed in this study are available from the corresponding author on reasonable request.

Human research participants

Policy information about studies involving human research participants and Sex and Gender in Research.

Reporting on sex and gender	Between June and Dec 2021, 49 lactating mothers planning on receiving BNT162b2 were recruited and followed up at four timepoints. Participants' ages ranged from 25 to 42 years, with a median age of 36 years (interquartile range (IQR): 31, 39).
Population characteristics	Nearly half (44.2%) of the participants gave birth via planned cesarean section. The intervals between two vaccine doses ranged between 20 – 42 days (median: 21 days, IQR: 21, 27).
Recruitment	We recruited 49 lactating mothers who planned to receive the BNT162b2 mRNA vaccine, when more than 99% of the Hong Kong population was not infected by COVID-19 (late 2021). Mothers who were serologically negative for SARS-CoV-2 pre-vaccination were included, while those who failed to receive two doses of BNT162b2 were excluded.
Ethics oversight	The study protocol was approved by the University of Hong Kong/Hospital Authority Hong Kong West Cluster Institutional review board (IRB Ref. No. UW-21-415). All participants provided written consent during enrolment.

Note that full information on the approval of the study protocol must also be provided in the manuscript.

Field-specific reporting

Please select the one below that is the best fit for your research. If you are not sure, read the appropriate sections before making your selection.

☒ Life sciences ☐ Behavioural & social sciences ☐ Ecological, evolutionary & environmental sciences

For a reference copy of the document with all sections, see nature.com/documents/nr-reporting-summary-flat.pdf

Life sciences study design

All studies must disclose on these points even when the disclosure is negative.

Sample size	To the best of our knowledge, this is the first longitudinal human study to show that baseline breast milk microbiota composition and its post-vaccination changes reflect vaccine-conferred anti-SARS-CoV-2 antibody levels in breast milk. There is no data regarding microbial diversity in breast milk from lactating mothers who received two dose mRNA SARS-CoV-2 vaccine. Moreover, it is hard to recruit subjects during the pandemic and the vaccination hesitancy. As such, we did not estimate the sample size strictly . Nevertheless, this limitation was made up through including an external validation cohort, albeit samples were collected pre-pandemic.
Data exclusions	Those who failed to receive two doses of BNT162b2 were excluded. After removing samples without available sequencing data and one outlier (positive ELISA result pre-vaccination), 175 samples from 44 participants remained for the data analyses.
Replication	NA.
Randomization	NA. This study is a prospective cohort study, which is insufficient for treatment randomization.
Blinding	NA. This study is a prospective cohort study, which is insufficient for blinding

Reporting for specific materials, systems and methods

We require information from authors about some types of materials, experimental systems and methods used in many studies. Here, indicate whether each material, system or method listed is relevant to your study. If you are not sure if a list item applies to your research, read the appropriate section before selecting a response.

Materials & experimental systems

n/a	Involved in the study
☒	☐ Antibodies
☒	☐ Eukaryotic cell lines
☒	☐ Palaeontology and archaeology
☒	☐ Animals and other organisms
☐	☒ Clinical data
☒	☐ Dual use research of concern

Methods

n/a	Involved in the study
☒	☐ ChIP-seq
☒	☐ Flow cytometry
☒	☐ MRI-based neuroimaging

Clinical data

Policy information about clinical studies

All manuscripts should comply with the ICMJE guidelines for publication of clinical research and a completed CONSORT checklist must be included with all submissions.

Clinical trial registration: This study is a prospective cohort study, which has not been yet registered. This study followed the STROBE reporting guideline.

Study protocol: NA

Data collection: In total of 49 lactating mothers who planned to receive the BNT162b2 mRNA vaccine, when more than 99% of the Hong Kong population was not infected by COVID-19. Breast milk was self-collected at the participants' home in sterile milk bags at four timepoints (pre-vaccination, one week post-first dose, one week post-second dose, and one month post-second dose).

Outcomes: Immune responses to BNT162b2, including SARS-CoV-2 spike protein-specific IgA and IgG levels and microbiota abundances in breast milk at different timepoints, were regarded as the main outcome. SARS-CoV-2 spike protein-specific IgA and IgG levels in breast milk were measured to assess immune responses to BNT162b2 at different stages of the vaccination regimen using ELISA. DNA was extracted from breast milk using QIAamp PowerFecal Pro DNA Kit (QIAGEN, Hilden, Germany). V3-V4 regions of the 16S rRNA genes were amplified and sequenced by Illumina MiSeq.
